# Supplementary material for: A Literature Review and Gap Analysis of Emerging Technologies and New Trends in Gambling
Source: Int J Environ Res Public Health. 2020 Jan 23;17(3):744. doi: 10.3390/ijerph17030744 (PMC7036923; doi:10.3390/ijerph17030744)
Supplement: Supplementary file 1 [file ijerph-17-00744-s001.pdf]

Table S1. Quality Ratings for Peer-reviewed Literature—Technology and Trends (Qualitative).

	Author																		
**Rating Criteria**	**Abarbanel (2017)**	**Armstrong (2016)**	**Deans (2016)**	**Deans (2016)**	**Gainsbury (2015)**	**Gainsbury King (2015)**	**Gainsbuy Delfabbro (2016)**	**Goodwin (2017)**	**Hing (2015)**	**Hing, Sproston (2017)**	**Kim (2016)**	**Kim (2017)**	**Kristiansen (2017)**	**Landon (2016)**	**Lopez-Gonzales (2018)**	**Pitt (2017)**	**Pitt (2017)**	**Stead (2016)**	**Talberg (2017)**
1. Is the qualitative approach appropriate to answer the research question?	Y	Y	Y	Y	Y	Y	Y	Y	Y	Y	Y	Y	Y	Y	Y	Y	Y	Y	Y
2. Are the qualitative data collection methods adequate to address the research question?	Y	Y	Y	Y	Y	Y	Y	Y	Y	Y	Y	Y	Y	Y	Y	Y	Y	Y	Y
3. Are the findings adequately derived from the data?	Y	Y	Y	Y	Y	Y	Y	Y	Y	Y	Y	Y	Y	Y	Y	Y	Y	Y	Y
4. Is the interpretation of results sufficiently substantiated by data?	Y	Y	Y	Y	Y	Y	Y	Y	Y	Y	Y	Y	Y	Y	Y	Y	Y	Y	Y
5. Is there coherence between qualitative data sources, collection, analysis and interpretation?	Y	Y	Y	Y	Y	Y	Y	Y	Y	Y	N	Y	Y	Y	Y	Y	Y	Y	Y

Table S2. Quality Ratings for Peer-reviewed Literature—Technology and Trends (Quant RCTs).

Rating Criteria	Author				
	**Li (2016)**	**O'Loughlin (2018)**	**Petry (2015)**	**Quignmo (2016)**	**Riva (2015)**
1. Is randomization appropriately performed?	Y	Y	Y	Y	Y
2. Are the groups comparable at baseline?	Y	Y	Y	Y	Y
3. Are there complete outcome data?	Y	Y	Y	Y	Y
4. Are outcome assessors blinded to the intervention provided?	N/A	N/A	?	Y	Y
5. Did the participants adhere to the assigned intervention?	Y	Y	Y	Y	Y

Table S3. Quality Ratings for Peer-reviewed Literature—Technology and Trends (Quant Non-Randomised).

Rating Criteria	Author				
	**Donaldson (2016)**	**Frahn (2015)**	**Montes (2017)**	**Murch (2019)**	**Worhunsky (2018)**
1. Are the participants representative of the target population?	Y	Y	Y	Y	Y
2. Are measurements appropriate regarding both the outcome and intervention (or exposure)?	Y	Y	Y	Y	Y
3. Are there complete outcome data?	Y	Y	Y	Y	Y
4. Are the confounders accounted for in the design and analysis?	Y	Y	Y	Y	Y
5. During the study period, is the intervention administered (or exposure occurred) as intended?	Y	Y	Y	Y	Y

Table S4. Quality Ratings for Peer-reviewed Literature—Technology and Trends (Quant Descriptive).

Rating Criteria	Author																			
	**Abarbanel (2015)**	**Baggio (2016)**	**Baggio (2017)**	**Baloglu (2014)**	**Barrault (2016)**	**Biolcati (2015)**	**Blaszczynski (2016)**	**Browne (2015)**	**Canalle (2016)**	**Costes (2016)**	**d'Astous (2015)**	**Deans (2016)**	**Dussault (2017)**	**Dwyer (2018)**	**Dwyer (2018)**	**Effertz (2018)**	**Elton-Marshall (2016)**	**Estevez (2017)**	**Forrest (2016)**	**Gainsbury (2015) a**
1. Is the sampling strategy relevant to address the research question?	Y	Y	Y	Y	Y	Y	Y	Y	Y	Y	Y	Y	Y	Y	Y	Y	Y	Y	Y	Y
2. Is the sample representative of the target population?	Y	Y	Y	Y	Y	Y	Y	N	Y	Y	Y	Y	Y	Y	Y	Y	Y	Y	Y	Y
3. Are the measurements appropriate?	Y	Y	Y	Y	Y	Y	Y	Y	Y	Y	Y	Y	Y	Y	Y	Y	Y	Y	Y	Y
4. Is the risk of nonresponse bias low?	Y	Y	Y	Y	Y	Y	Y	Y	Y	Y	Y	Y	Y	Y	Y	Y	Y	Y	Y	Y
5. Is the statistical analysis appropriate to answer the research question?	Y	Y	Y	Y	Y	Y	Y	Y	Y	Y	Y	Y	Y	Y	Y	Y	Y	Y	Y	Y

Rating Criteria	Author																			
	Gainsbury (2015) b	Gainsbury (2015) c	Gainsbury (2015) d	Gainsbury (2015) e	Gainsbury (2016)	Gainsbury (2017)	Gainsbury (2018)	Gassman (2017)	Giotakos (2017)	Goldstein (2016)	Gray (2015)	Haefeli (2015)	Hayer (2018)	Hing (2016)	Hing, Myles (2017)	Hing, Russel l(2017)	Hing, Russell (2018)	Ho (2017)	Hollingshead (2016)	Kairouz (2018)
1. Is the sampling strategy relevant to address the research question?	Y	Y	Y	Y	Y	Y	Y	Y	Y	Y	Y	Y	Y	Y	Y	Y	Y	Y	Y	Y
2. Is the sample representative of the target population?	Y	Y	Y	Y	Y	Y	Y	Y	Y	Y	Y	Y	Y	Y	Y	Y	Y	Y	Y	Y
3. Are the measurements appropriate?	Y	Y	Y	Y	Y	Y	Y	Y	Y	Y	Y	Y	Y	Y	Y	Y	Y	Y	Y	Y
4. Is the risk of nonresponse bias low?	Y	Y	Y	Y	Y	Y	Y	Y	Y	Y	Y	Y	Y	Y	Y	Y	Y	Y	Y	Y
5. Is the statistical analysis appropriate to answer the research question?	Y	Y	Y	Y	Y	Y	Y	Y	Y	Y	Y	Y	Y	Y	Y	Y	Y	Y	Y	Y

Rating Criteria	Author																			
	Khazaal (2018)	Kim (2015)	King Delfabbro (2016)	Li (2015)	Lin (2015)	Lopez-Gonzalez (2018)	Macey (2018)	MacLaren (2016)	Marchica (2016)	Marchica (2017)	Martin (2016)	McBride (2016)	Molde (2018)	Mulkeen (2017)	Nower (2018)	Newall (2017)	Papineau (2018)	Pitt (2017)	Prentice (2015)	Quilty (2016)
1. Is the sampling strategy relevant to address the research question?	Y	Y	Y	Y	Y	Y	Y	Y	Y	Y	Y	Y	Y	Y	Y	Y	Y	Y	Y	Y
2. Is the sample representative of the target population?	Y	Y	Y	Y	Y	Y	Y	Y	Y	Y	N	Y	Y	Y	Y	Y	Y	Y	Y	Y
3. Are the measurements appropriate?	Y	Y	Y	Y	Y	Y	Y	Y	Y	Y	Y	Y	Y	Y	Y	Y	Y	Y	Y	Y
4. Is the risk of nonresponse bias low?	Y	Y	Y	Y	Y	Y	Y	Y	Y	Y	Y	Y	Y	Y	Y	Y	Y	Y	Y	Y
5. Is the statistical analysis appropriate to answer the research question?	Y	Y	Y	Y	Y	Y	Y	Y	Y	Y	Y	Y	Y	Y	Y	Y	Y	Y	Y	Y

Rating Criteria	Redono (2015)	Remond (2018)	Sanders (2018)	Sigerson (2017)	Teichert (2017)	Wann (2015)	Wijesingha (2015)	Yoo (2016)	Yuan (2015)	Zhang (2018)
1. Is the sampling strategy relevant to address the research question?	Y	Y	Y	Y	Y	Y	Y	Y	Y	Y
2. Is the sample representative of the target population?	Y	Y	Y	Y	Y	Y	Y	Y	Y	Y
3. Are the measurements appropriate?	Y	Y	Y	Y	Y	Y	Y	Y	Y	Y
4. Is the risk of nonresponse bias low?	Y	Y	Y	Y	Y	Y	Y	Y	Y	Y
5. Is the statistical analysis appropriate to answer the research question?	Y	Y	Y	Y	Y	Y	Y	Y	Y	Y

Table S5. Summary of Internet gambling articles.

Author(s) & Year/Study Location	Article Type	Aim	Key Findings	Gaps Identified By The Authors
Baggio et al. (2016)[1]<br><br>France & Switzerland	Cross-sectional research	To investigate the co-morbidity of problem gambling and generalised problem internet use among Internet and land-based gamblers	Problem gambling and problem internet use appeared as separate disorders, but their relationship was increased among internet gamblers in comparison with land-based gamblers	**Knowledge gap [More research in general]**<br>Inconsistent results regarding the associations between problem gambling and problem internet use needs further investigation
Baggio et al. (2017)[2]<br><br>France	Cross-sectional research	To test whether the relationship between Internet gambling and gambling problems persisted when including variables related to gambling involvement as predictors, namely time spent gambling and diversity of gambling formats	Internet gamblers had significantly more problems than land-based gamblers. However, the relationship decreased when diversity of gambling formats and time spent gambling were controlled separately, becoming non-significant when both were included in the model	**Method gaps [Longitudinal research; Use of improved outcome measures]**<br>Longitudinal studies are needed to understand the causal paths between Internet gambling, gambling involvement, and severity of problem gambling;<br>Studies including clinical assessment of disordered gambling may be used in further studies, as well as more precise measures of number of gambling formats and time spent gambling<br><br>**Knowledge gaps [Sub-populations]**<br>Further studies with subsamples of heavy gamblers are needed to investigate the importance of time spent gambling;<br>Groups of only-Internet gamblers, exclusively land-based gamblers, and both Internet and land-based gamblers should be assessed to investigate whether there were differences between being only an Internet gambler or using both the Internet and land-based venues
Barrault & Varescon (2016)[3]	Cross-sectional research	To assess sociodemographic characteristics, gambling practice and impulsive	Regular players were young men, executives or students, mostly single and working full-time. Online players played significantly more often	**Knowledge gaps [Research on specific factors: Psychological]**<br>Investigation of motivations to engage in live or online gambling is needed;

France		sensation seeking among a population of regular poker players with different levels of gambling intensity and to compare online and live players.	whereas live players reported significantly longer gambling sessions. Sensation seeking was high across all groups, whereas impulsivity significantly distinguished players according to the intensity of gambling	Further research should investigate the links between impulsive sensation seeking and normal and pathological poker playing more closely;<br>Further research into the respective weight of cognitive distortions and negative mood states is needed
Biolcati et al. (2015)[4]<br><br>Italy	Cross-sectional research	To investigate: (i) the playing motivations of recreational and professional Texas Hold'em poker players, (ii) the psychological characteristics of online poker players in relation to narcissism, impulsivity, self-esteem, and problem gambling, and (iii) the differences between professional and recreational poker player on these psychological variables	Only 1.6% poker players met the DSM-IV-TR diagnostic criteria for pathological gambling. Generally, poker players had low scores on *narcissism* and *impulsivity*, and high scores on *self-esteem*	**Knowledge gaps [Research on specific factors: Psychological]**<br>Future studies should analyse online poker players' relationship styles, sense of loneliness, agreeableness etc<br><br>**Knowledge gap [Sub-populations]**<br>More studies are needed to investigate control populations to directly compare the Texas Hold'em online poker player with the general population as well as other gamblers
Blaszczynski et al. (2016)[5]<br><br>Australia	Cross-sectional research	To investigate differences in mental health status in exclusive online, exclusive land-based, and mixed Internet and land-based samples of gamblers drawn from the general population	Mixed gamblers exhibited higher scores on the Problem Gambling Severity Index, more gambling involvement and more alcohol consumption than exclusive online gamblers. Land-based gamblers experienced higher levels of psychological distress, self-acknowledged need for treatment, and help-seeking behaviour	**Knowledge gap [Sub-populations]**<br>Understanding the characteristics of different problem gambling subpopulations may inform the development of more effective targeted interventions<br><br>**Method gap [Longitudinal research]**<br>Longitudinal studies are needed to unravel causal relationships implicated by the findings
Canale et al. (2016)[6]<br><br>Italy	Cross-sectional research	To understand the impact of online gambling on gambling problems in a large-scale nationally representative sample of Italian youth, and to identify and then further examine a subgroup of online gamblers who reported higher rates of gambling problems	Rates of problem gambling were five times higher among online gamblers than non-online gamblers. Factors that increased the risk of becoming a problem online gambler included living with non-birth parents, having a higher perception of financial family status, being more involved with gambling, and the medium preferences of remote gamblers (e.g., Internet cafes, digital television, and video game console)	**Knowledge gap [Sub-populations]**<br>Future studies should quantify the extent of involvement in online gambling to enable evaluation of regular Internet gambling;<br>The comparisons between the more nuanced groups of gamblers (offline gambling only, online gambling only, online and offline gambling but on different activities, online and offline gambling but on the same activities) warrant additional study<br><br>**Knowledge gap [Research on specific factors: Psychological]**<br>Future studies may benefit from including impulsivity traits and reasons for gambling to better elucidate the differences between online and non-online gamblers
Chagas & Gomes (2017)[7]<br><br>-	Critical review of the literature	To review and analyse studies that are focused on Internet gambling with the use of behavioural tracking and big data to identify gambling behaviour	The body of published research on real-world Internet gambling behaviour is limited; however, there is a trend in the industry to make data available to researchers.<br>Studies have essentially been conducted with data from European and Australian players	**Knowledge gap [Replication in other locations]**<br>Future studies should be conducted in locations other than Australia and Europe<br><br>**Knowledge gap [Research on specific factors: Gambling intentions/behaviours/actions]**

				Future research might include risk factors and the assessment of trends of player spending, deposits, losses, changes in player funding sources, and gambling behaviour related to time spent gambling;<br><br>**Method gap [Integration between studies]**<br>Another direction for future research is the integration of findings from studies of behavioural tracking with self-report data<br><br>**Public health/practical knowledge gap [Responsible gambling]**<br>Further research on safer gaming parameters and self-exclusion is needed<br><br>**Knowledge gap [Research on specific factors: Psychological]**<br>There is a need to better understand players' motivations<br><br>**Knowledge gap [Other/related technologies/trends]**<br>Additional avenues for future research are the convergence of gaming and gambling, the use of virtual currencies and monetisation
Costes et al. (2016)[8]<br><br>France	Cross-sectional research	To assess differences in gambling patterns and related harm between online gamblers who use licensed versus unlicensed sites	53.7% of online gamblers reported gambling exclusively on licensed sites. Characteristics of those who bet on regulated activities on unlicensed sites were: female, younger, less educated, inactive in the labour market and more likely to perceive their financial situation to be difficult. Gambling on unlicensed sites was also associated with more intense gambling patterns and more gambling-related problems	**Public health/practical knowledge gap [Gambling regulation]**<br>Future research should consider expanding analysis (e.g., combining multiple data sources) to gain a more comprehensive understanding of the effectiveness of regulating gambling activities in protecting vulnerable gambler
d'Astous & Gaspero (2015)[9]<br><br>France & Canada	Cross-sectional, mixed-methods research	To examine the occurrence of heuristic (i.e., intuitive and fast) and analytic (i.e., deliberate and slow) processes among people who engage in online sports betting on a regular basis	Results showed that heuristic and analytic processes act as mediators of the relationship between experience and performance	**Knowledge gaps [Applicability to other populations; Applicability to other gambling types]**<br>More research is needed to confirm the study findings and explore the applicability of the of the dual mediation process model in other populations and types of gambling
Deans et al. (2016)[10]<br><br>Australia	Qualitative research	To explore the ways in which online and land-based environments influence gambling risk behaviours	Online factors: globalisation of betting markets and options, easy access via mobile, virtual nature of amounts spent and lost, industry inducements<br>Land-based factors: provide a conducive atmosphere- televised sports, multiple forms of gambling, supply of alcohol, cash in hand wins, exciting social atmosphere, 'a male gambling subculture', gambling promotions	**Knowledge gap [More research in general]**<br>Future studies should keep exploring contextual factors on a range of bettors
Effertz et al. (2018)[11]	Cross-sectional research	To estimate the effect of online gambling on gambling	On average, replacing 10% of offline gambling with online gambling increases the likelihood of being a problematic gambler by 8.8-12.6%. This increase is	**Knowledge gap [More research in general]**<br>Findings need to be tested using more recent data

Germany		problems and resulting economic health costs	equivalent to 139, 322 problematic gamblers and 27.24 million euro per year of additional expenditures in the German health sector	**Knowledge gap [Research on specific factors: Gambling outcomes]** Future research should include social and/or economic cost of gambling as opposed to the treatment cost only
Elton-Marshall et al. (2016)[12] Canada	Cross-sectional research	To examine online and land-based gambling behaviour among adolescents in 3 Canadian provinces prior to the implementation of legalised online gambling	Adolescents were engaging in online gambling at a significantly higher rate than has been previously found and were using technology to engage in simulated gambling	**Knowledge gap [Research on specific factors: Gambling intentions/behaviours/actions]** Further research is needed to understand whether adolescents are betting on the outcomes of video games or engaging in video games that include gambling for money, or both; Further research is needed to identify which websites youth are accessing
Estevez et al. (2017)[13] Spain	Cross-sectional research	To examine the differences between adults with gambling disorder who exclusively make sports bets online, those that are non-sports internet gamblers, and offline gamblers	No differences in terms of gambling severity were identified between groups. However, gambling disorder patients who exclusively bet online appeared to possess distinct personality characteristics (e.g. persistence) and higher debt levels compared with offline gamblers.	**Method gap [More representative samples/other sampling gaps]** Future research should include stronger study designs with larger, and more balanced samples to verify the study findings
Gainsbury & Russell (2015)[14]	Cross-sectional research	To investigate online wagering patterns by analysing account data from an Australian corporate bookmaker	The majority of bets placed were for a win (45.31%) and were placed on races (86.74%) or sports (11.29%), and 77.63% of the bets were losses. Overall, wagering was deemed as an entertainment activity, and the majority of customers were motivated by factors other than simply winning money	**Method gap [Use of improved outcome measures]** Future research should specifically compare betting patterns and outcomes for wagering and gaming (casino games and slot machines)
Gainsbury, Russell, Blaszczynski & Hing (2015)[15] Australia	Cross-sectional research	To examine differences between Internet gamblers with a single or multiple online gambling accounts, including their gambling behaviours, factors influencing their online gambling and risk of experiencing gambling problems	Results indicate two types of gamblers - one motivated to move between sites to optimize preferred experiences with a tendency to gamble in a more volatile manner; and a smaller, but more stable group less influenced by promotions and experiences, and seeking a reputable and safe gambling experience	**Knowledge gap [Research on specific factors: Gambling intentions/behaviours/actions]** Further research is needed to explore how Internet gamblers use multiple sites and their motivations for doing so
Gainsbury, Russell, Blaszczynski & Hing (2015)[16] Australia	Cross-sectional research	To investigate subgroups of gamblers (Internet-only (IG), land-based only (LBG) or mixed-mode (MMG)) to identify the potential harms associated with various forms and modes of gambling	Significant socio-demographic differences between groups were found, with the LBGs being older and MMGs the younger. MMGs engaged in the greatest variety of gambling, most likely to gamble frequently on sports and races. LBGs had a higher proportion of problem gamblers than IGs and more likely to play EGM weekly.	**Knowledge gap [More research in general]** Further research is needed to explore the interaction between forms and modes of gambling to understand the risk of problem gambling<br><br>**Knowledge gap [Sub-populations]** A non-internet sample and mode of survey is needed for future studies to include all sorts of users
Gainsbury, Russell, Wood, Hing & Blaszczynski (2015)[17] Australia	Cross-sectional research	Compared problem with non-problem and at-risk Internet gamblers to understand why some Internet gamblers experience gambling-related harms	Problem gambling respondents were younger, less educated, higher household debt, lost more money, gambled on a greater number of activities, and were more likely to use drugs than non-problem and at-risk gamblers. Problem gamblers also had more irrational beliefs about gambling. Internet problem	**Knowledge gap [Sub-populations]** Future research should concentrate on a more involved sample of internet gamblers

			gamblers in particular disclosed problems with sleeping and eating	
Gainsbury, Russell, Hing, Wood, Lubman & Blaszczynski (2015)[18]<br><br>Australia	Cross-sectional research	To investigate the prevalence of gambling among Australian adults and the relationship between various gambling activities and interactive modes of access	This study concluded that the nature of gambling participation is shifting with more having interactive gambling, specially males and younger population	**Method gap [More representative sample/other sampling gaps]**<br>Future research is needed to address the limitations of the data resulting from sampling only participants with landline telephones
Gainsbury et al. (2016)[19]<br><br>Australia	Cross-sectional research	Used propensity score matching, to estimate the consequence of gambling offline, or online through a computer, as compared to mobile or other supplementary devices	Online computer gamblers had lower gambling problems compared to those used mobile or supplementary devices. Age, marital and employment status were predictors of how people gambled online	**Knowledge gap [More research in general]**<br>Further research is needed examining the relation between technological access points and gambling harms
Gainsbury, Abarbanel & Blaszczynski (2017)[20]<br><br>Australia	Cross-sectional research	To describe the characteristics of Australian online esports gamblers as compared to those wagering on sports online	Esports bettors have a higher proportion of females, individuals with Asian ethnic background, young, and with higher household income compared to sports bettors. This is quite different from white males that are typically featured in betting advertisements. Esports bettors were also more frequent gamblers, used greater variety of devices, were more likely to use offshore sites, and likely early technology adopters.	**Knowledge gap [Research on specific factors: Psychological]**<br>Future research should try to understand the motivations for online gambling among different cohorts<br><br>**Knowledge gap [More research in general]**<br>As a relatively new area of gambling, more research is needed on Esports
Gainsbury et al. (2018)[21]<br><br>Australia	Cross-sectional research	To explore the extent to which online gamblers use offshore as compared to domestic gambling sites	Only 25.8% of online gamblers used offshore sites, and they were more involved with gambling featuring greater problem gambling severity	**Knowledge gap [Research on specific factors: Gambling outcomes]**<br>Further research is needed to understand the specific harms experienced in relation to the use of offshore gambling sites<br><br>**Knowledge gap [Research on specific factors: Gambling intentions/behaviours/actions]**<br>Research is needed to understand the features and signals that consumers use to identify a gambling site as domestically licensed as compared to offshore, or other features used to indicate a 'safe' site
Giotakos et al. (2017)[22]<br><br>Greece	Cross-sectional research	To evaluate the association of Internet gambling with Internet addiction, online sexual engagement, suicidality and substance use, in a sample of Greek adults	Internet addiction significantly predicted engagement with online gambling, followed by substance use in general, and in particular, the use of cocaine or heroin	**Method gap [Use of data mining]**<br>Further research is needing using more sophisticated methods such as data mining<br><br>**Knowledge gap [Research on specific factors: Gambling intentions/behaviours/actions]**<br>Research is needed co-examining latent gambling (dealing with the stock market) and their interaction with online and conventional gambling practices

Goldstein et al. (2016)[23]<br><br>Canada	Cross-sectional research	To examine differences between young adult online and non-online gamblers	Males, baseline coping motives for gambling and negative affect emerged as significant correlates of online gambling. Online gamblers scored higher on a baseline measure of pathological gambling and spent more money over the 30 days. Non-online gamblers on the other side, consumed more alcohol while gambling. Online gambling was more often initiated for personal reasons whereas non-online gambling was often initiated for social reasons	**Method gap [More representative samples/other sampling gaps; Use of improved outcome measures]**<br>Future research should use more representative sample and validated measures to confirm these findings
Gray et al. (2015)[24]<br><br>Iceland	Longitudinal research	To examine patterns of gambling behaviour among a cohort of Icelandic residents who subscribed to I´slensk Getspa´ during January 2010	A typical subscriber bet approximately 3 days per month and made fewer than two bets per gambling day, each worth approximately the equivalent of USD 4. Subscribers lost the bulk (96%) of the amount they wagered, for a total loss of approximately USD 40 across the 2-year window of observation	**Method gap [Use of improved outcome measures]**<br>Further research should triangulate on a fuller description of problematic Internet gambling using a combination of betting records and self-report measures
Haefeli et al. (2015)[25]<br><br>Austria	Exploratory research	A sample of 1008 emails from self-excluders and controls to the customer services of an online gambling operator was reanalysed to explore the possibility of using automated text analysis software to extract quantitative markers from written player correspondence	Results indicated that automated text analysis could be deployed as an expert system to prioritize cases and to support human judgement	**Knowledge gap [More research in general]**<br>The findings need to be confirmed using more recent data and by comparing with other market data
Hing et al. (2015)[26]<br><br>Australia	Qualitative research	To explore the psychosocial factors and processes related to maintaining and losing control during internet gambling	The most frequently identified aspects of Internet gambling leading to impaired control were use of digital money, access to credit, lack of scrutiny and ready accessibility. Participants used arrange of self-limiting strategies with variable success and suggested that more comprehensive RG measures were required of internet gambling operators	**Method gap [Use of data mining]**<br>Further research is needed to more accurately determine the effect of various features of internet gambling, ideally using real player data from Internet gambling operators
Hing et al. (2017)[27]<br><br>Australia	Cross-sectional research	To: (1) determine demographic, behavioural and psychological risk factors for gambling problems on online EGMs, online sports betting and online race betting; (2) compare the characteristics of problematic online gamblers on each of these online forms	Key risk factors for online EGM gambling were: more frequent play on online EGMs, substance use when gambling, and higher psychological distress. For both online sports and race betting: being male, younger, speaking a language other than English, more frequent betting and more negative attitude toward gambling were commonly identified risk factors. Additionally, identified factors were: a) higher psychological stress for online sports betting; and b) engagement with more gambling forms, self-reported semi-professional/ professional and illicit drug use whilst gambling for online race betting	**Knowledge gaps [Sub-populations]**<br>Further research is needed to ascertain whether substance use amongst problem gamblers is more frequently associated with online compared to land-based EGM gambling;<br>Further research is needed on the ethnic profiles of problematic online sports bettors and race bettors

Ho (2017)[28]<br><br>China	Cross-sectional research	To investigate the relationships between types of gambling activity, problem behaviours, and self-esteem with adolescent problem gambling in Hong Kong	Playing Poker and gaming in Amusement Game Centers had the strongest predictive value, implying a gambling-permissive culture regarding gambling as an entertainment, with parental and societal support and availability of gambling opportunities, has more impact on adolescent problem gambling than other personal risk factors like problem behaviours and low self-esteem	**Method gap [More representative samples/other sampling gaps]**<br>Large-scale research with a random sample is needed so that the predictors of adolescent problem gambling can be further examined<br><br>**Knowledge gaps [Research on specific factors: Psychological]**<br>Further research is needed focusing on factors affecting self-esteem so that concerned parties can make a concerted effort in enhancing adolescents' positive development
James et al. (2017)[29]<br><br>-	Narrative review	The review looks at mobile gambling and whether this emerging platform has distinguishing psychological features that may be particularly risky to gamblers, either a subset of current gamblers or a new population of gamblers	The authors conclude schedules of reinforcement found in gambling interact with the ways in which people tend to use smartphones that may expedite the acquisition of maladaptive learned behaviours such as problem gambling	**Knowledge gap [More research in general]**<br>Further research is needed exploring the potential for mobile gambling games to provide a user experience that is different from online games
Kairouz et al. (2018)[30]<br><br>Canada	Longitudinal research	To examine gambling patterns before and after legalisation of online gambling	The prevalence of internet gambling remained stable: 1.5% of the population gambled online in 2012 compared to 1.4% in 2009. Of those surveyed, 82.5% continued to gamble on unregulated sites in 2012 and data from OPD-UHH confirmed that 90% of all real money online poker players still bet on unregulated sites in 2013	**Method gap [Longitudinal research]**<br>Further longitudinal research is needed to disentangle the effects of legalisation of online gambling
Khazaal et al. (2017)[31]<br><br>Switzerland	Cross-sectional research	To characterize online gamblers in relation to indebtedness, loneliness, and several in-game social behaviours	Three clusters were found: lonely indebted (C1: 6.5%), not lonely not indebted (C2: 75.4%), and not lonely indebted gamblers (C3:18%). Participants of C1 were particularly at higher risk of problem gambling. The three groups differed on most assessed outcomes except for sensation seeking sub-score	**Knowledge gap [Research on specific factors: Gambling intentions/behaviours/actions]**<br>Further research is needed on the social aspects of online gambling and in-game social interactions
Kristiansen & Trajberg (2017)[32]<br><br>Denmark	Qualitative research	To explore how young people experience and respond to changes in gambling opportunities	Four overarching themes were identified: legislation, conditions of entry, proximity and technological innovations.	**Method gap [Qualitative research]**<br>Further research is needed:<br>To shed light on the dimensions of the online interaction between adolescents and gambling operators;<br>To assess whether money-free online gambling encourages positive attitudes towards commercial gambling, and the effects of online advertising on young people, including minors;<br>To explore perceptions and implications of free bonuses
Montes & Weatherly (2017)[33]<br><br>USA	Comparative study with controls	To examine the differences in gambling behaviour of online and non-online gamblers in a	Online gamblers engaged in potentially more deleterious gambling behaviour than non-online gamblers	**Method gap [Experimental research]**<br>An examination of the gambling behaviour of self-reported online and non-online student gamblers in a controlled laboratory environment is needed

		controlled, laboratory environment		
Mulkeen et al. (2017)[34]<br><br>UK	Cross-sectional research	To explore consumer perceptions of responsible gambling by evaluating players' perceptions of motives to gamble online and their opinions relating to gambling practices and behavioural factors that enable them to gamble in a responsible and ethical way	Most significant factors perceived by players were escape and relaxation; financial motivation; social and competition. In terms of player views in relation to responsible gambling practices and behavioural factors both self-exclusion and self-help; and game design are identified as the key factors. Financial motive to gamble were divided into the following sub-motives: 'to win money' and to 'earn income'	**Knowledge gap [Research on specific factors: Psychological]**<br>Future research on autonomy and mastery is needed;<br><br>**Public health/practical knowledge gap [Responsible gambling]**<br>The effectiveness of self-regulation needs further exploration
Papineau et al. (2018)[35]<br><br>Canada	Cross-sectional research	To assess the differential impacts of online, mixed, and offline gambling	Online gambling resulted in an extra burden of impacts in several aspects of lives: work, relationship, mental/physical health, finances etc. Combined with offline gambling, online gambling significantly increased the burden of impacts in terms of both the number and intensity of impacts	**Knowledge gap [Research on specific factors: Gambling intentions/behaviours/actions]**<br>More detailed analyses of the impact of different gambling platforms are needed
Petry & Gonzalez-Ibanez (2015)[36]<br><br>USA	Randomised Controlled Trial	To evaluate a brief intervention to reduce gambling in Internet and non-internet college student problem gamblers	Recent Internet gamblers demonstrated similar reductions in gambling over time and in response to the brief interventions as non-Internet gamblers	**Method gaps [Longitudinal research]**<br>Studies incorporating long-term follow-ups are needed to examine the sustainability of brief interventions
Redondo (2015)[37]<br><br>Spain	Cross-sectional research	To assess the risks associated with online lottery and casino gambling	Online lottery/casino players, compared to their offline counterparts, hade more psychographic risk factors such as a higher level of trust in the internet, which could increase susceptibility to unscrupulous manipulation	**Knowledge gap [Research on specific factors: Psychological]**<br>More research is needed exploring variables such as tendency towards escapism, attitude to risk, capacity for self-control
Remond & Romo (2018)[38]<br><br>France	Cross-sectional research	To investigate the intricacies between the player interface proposed by screens (in particular on smartphone applications or in video games) and gambling	Findings suggested immersion variables made it possible to understand the cognitive participation of individuals towards screens in general, the practice of gambling on screens and the excessive practice of screens	**Method gaps [Longitudinal research]**<br>**Knowledge gap [Research on specific factors: Psychological]**<br>Future studies should incorporate neuropsychological measurements and longitudinal analysis to allow a more precise glimpse of the cognitive processes related to immersion variables
Sigerson et al. (2017)[39]<br><br>USA	Cross-sectional research	To investigate the relationships of information technology addictions with other behavioural addictions	The spectrum approach conceptualised information technology addiction as a cluster of disorders comprising not only shared risk factors and symptoms but also distinct characteristics. Information technology is more similar to other behavioural addictions than substance-related addictions	**Knowledge gap [More research in general]**<br>Future research should study information technology addictions and other behavioural addictions together, and explore the commonalities and differences among them
Stead et al. (2016)[40]<br><br>UK	Qualitative research	To investigate the appeal of online bingo	Comparison of website content with participants' reasons to play bingo showed congruence between the strategies used by the bingo websites and the motivations of bingo players themselves and the	**Public health/practical knowledge gap [Gambling regulation]**<br>**Knowledge gap [Research on specific factors: Gambling outcomes]**

			benefits which they seek; suggesting that bingo websites strive to replicate and update the sociability of traditional bingo halls	Future research is needed to understand how the online bingo industry is regulated, as well as the effects of online bingo on individual gambling 'careers'
Talberg (2017)[41] Norway	Qualitative research	Twelve young poker players and three ''old-timers'' were interviewed about changes in online poker and problems with combining poker and education	Prioritizing between poker and education could be understood in terms of a weight balance (income from poker). Since the current poker population are more skilful, the games have become less profitable even for the best players and thus, reduce a student's inclination to drop out of education	**Knowledge gap [Research on specific factors: Psychological]** More research on poker players' learning processes and on variety of learning methods are needed
Wijesingha et al. (2017)[42] Canada	Cross-sectional research	To examine factors associated with online gambling for adolescents and compare this to land-based gamblers to examine whether the profile of online gamblers were unique	Adolescents being males, with any problem gambling severity (low to high) and who played free games, were significantly more likely to gamble online than those who did not have a gambling problem	**Public health/practical knowledge gap [Gambling regulation]** Future research should monitor the impact of recent online gambling legalization on adolescents
Yuan (2015)[43] China	Cross-sectional research	To examine the rationality of Chinese lottery gamblers	Chinese online lottery gamblers were significantly more likely to join a lottery package if it was proposed by proposers with higher return rates, suggesting that this population showed 'irrational' lottery gambling behaviour	**Knowledge gap [Research on specific factors: Psychological]** Future research should examine the reasons behind online lottery gamblers' 'irrational' behaviour, and its monetary consequence
Zhang et al. (2018)[44] Singapore	Cross-sectional research	To explore the harm associated with online gambling	The majority of the participants were male, aged under 30 (48%), and of Chinese ethnicity. The median largest ever debt and loss incurred as a result of online gambling was significantly larger than that due to offline. 18.4% participants waited between 1 to 2 years from their first online gambling experience to seek treatment and 17.3% waited for more than 10 years	**Method gaps [Longitudinal research]** Cohort studies are needed to determine longitudinal progression and outcomes

Internet gambling references

1. Baggio, S.; Gainsbury, S.M.; Berchtold, A.; Iglesias, K. Co-morbidity of gambling and Internet use among Internet and land-based gamblers: Classic and network approaches. *Int Gambl Stud* **2016**, *16*(3), 500-517.
2. Baggio, S.; Dupuis, M.; Berchtold, A.; Spilka, S.; Simon, O.; Studer, J. Is gambling involvement a confounding variable for the relationship between Internet gambling and gambling problem severity? *Computers Human Behav* **2017**, *71*, 148-152
3. Barrault, S.; Varescon, I. Online and live regular poker players: Do they differ in impulsive sensation seeking and gambling practice? *J Behav Addict* **2016**, *5*(1), 41-50.
4. Biolcati, R.; Passini, S.; Griffiths, M.D. All-in and bad beat: Professional poker players and pathological gambling. *Int J Ment Health Ad* **2015**, *13*(1), 19-32.
5. Blaszczynski, A.; Russell, A.; Gainsbury, S.M.; Hing, N. Mental health and online, land-based and mixed gamblers. *J Gambl Stud* **2016**, *32*, 261-275.
6. Canale, N.; Griffiths, M.D.; Vieno, A.; Siciliano, V.; Molinaro, S. Impact of Internet gambling on problem gambling among adolescents in Italy: Findings from a large-scale nationally representative survey. *Computers Human Behav* **2016**, *57*, 99-106.
7. Chagas, B.T.; Gomes, J.F. Internet gambling: A critical review of behavioural tracking research. *J Gambl Iss* **2017**, *36*, 1-27.
8. Costes, J-M.; Kairouz, S.; Eroukmanoff, V.; Monson, E. Gambling patterns and problems of gamblers on licensed and unlicensed sites in France. *J Gambl Stud* **2016**, *32*(1), 79-91.
9. d'Astous, A.; Di Gaspero, M. Heuristic and analytic processing in online sports betting. *J Gambl Stud* **2015**, *31*(2), 455-470.

10. Deans, E.G.; Thomas, S.L.;Daube, M.; Derevensky, J. "I can sit on the beach and punt through my mobile phone": The influence of physical and online environments on the gambling risk behaviours of young men. *Soc Sci Med* **2016**, *166*, 110-119.
11. Effertz, T.; Bischof, A.; Rumpf, H-J.; Meyer, C.; John, U. The effect of online gambling on gambling problems and resulting economic health costs in Germany. *Eur J Health Econ* **2018**, *19*(7), 967-978.
12. Elton-Marshall, T.; Leatherdale, S.T.; Turner, N.E. An examination of internet and land-based gambling among adolescents in three Canadian provinces: results from the youth gambling survey. *BMC Public Health* **2016**, *16*, 277.
13. Estevez, A.; Rodriguez, R.; Diaz, N.; Granero, R.; Mestre-Bach, G.; Steward, T.; Fernandez-Aranda, F.; Aymami, N.; Gomez-Pena, M.; del Pino-Gutierrez, A.; Bano, M.; Moragas, L.; Mallorqui-Bague, N.; Lopez-Gonzalez, H.; Jauregui, P.; Onaindia, J.; Martin-Romera, V.; Menchon, J.M.; Jimenez-Murcia, S. How do online sports gambling disorder patients compare with land-based patients? *J Behav Addict* **2017**, *6*(4), 639-647.
14. Gainsbury, S.M.; Russell, A. Betting patterns for sports and races: a longitudinal analysis of online wagering in Australia. *J Gambl Stud* **2015**, *31*(1), 17-32.
15. Gainsbury, S.M.; Russell, A.; Blaszczynski, A.; Hing, N. Greater involvement and diversity of Internet gambling as a risk factor for problem gambling. *Eur J Public Health*, **2015**, *25*(4), 723-728.
16. Gainsbury, S.M.; Russell, A.; Blaszczynski, A.; Hing, N. The interaction between gambling activities and modes of access: a comparison of Internet-only, land-based only, and mixed-mode gamblers. *Addict Behav* **2015**, *41*, 34-40.
17. Gainsbury, S.M.; Russell, A.; Wood, R.; Hing, N.; Blaszczynski, A. How risky is Internet gambling? A comparison of subgroups of Internet gamblers based on problem gambling status. *New Media Society* **2015**, *17*(6), 861-879.
18. Gainsbury, S.M.; Russell, A.; Hing, N.; Wood, R.; Lubman, D.; Blaszczynski, A. How the Internet is changing gambling: findings from an Australian Prevalence Survey. *J Gambl Stud* **2015**, *31*(1), 1-15.
19. Gainsbury, S.M.; Liu, Y.; Russell, A.M.T.; Teichert, T. Is all Internet gambling equally problematic? Considering the relationship between mode of access and gambling problems. *Computers Human Behav* **2016**, *55*, 717-728.
20. Gainsbury, S.M.; Abarbanel, B.; Blaszczynski, A. Game on: comparison of demographic profiles, consumption behaviors, and gambling site selection criteria of esports and sports bettors. *Gaming Law Rev* **2017**, *21*(8), 575-587.
21. Gainsbury, S.M.; Russell, A.M.; Hing, N.; Blaszczynski, A. Consumer engagement with and perceptions of offshore online gambling sites. *New Media Society* **2018**, *20*(8), 2990-3010.
22. Giotakos, O.; Tsouvelas, G.; Spourdalaki, E.; Janikian, M.; Tsitsika, A.; Vakirtzis, A. Internet gambling in relation to Internet addiction, substance use, online sexual engagement and suicidality in a Greek sample. *Int Gambl Stud* **2017**, *17*(1), 20-29.
23. Goldstein, A.L.; Vilhena-Churchill, N.; Stewart, S.H.; Hoaken, P.N.S.; Flett, G.L. Mood, motives, and money: An examination of factors that differentiate online and non-online young adult gamblers. *J Behav Addict* **2016**, *5*(1), 68-76.
24. Gray, H.M.; Jonsson, G.K.; LaPlante, D.A.; Shaffer, H.J. Expanding the study of internet gambling behavior: trends within the Icelandic lottery and sportsbetting platform. *J Gambl Stud* **2015**, *31*(2), 483-499.
25. Haefeli, J.; Lischer, S.; Haeusler, J. Communications-based early detection of gambling-related problems in online gambling. *Int Gambl Stud* **2015**, *15*(1), 23-38.
26. Hing, N.; Cherney, L.; Gainsbury, S.M.; Lubman, D.I.; Wood, R.T.; Blaszczynski, A. Maintaining and losing control during internet gambling: A qualitative study of gamblers' experiences. *New Media Society* **2015**, *17*(7), 1075-1095.
27. Hing, N.; Russell, A.M.; Browne, M. Risk factors for gambling problems on online electronic gaming machines, race betting and sports betting. *Front Psychol* **2017**, *8*, 779.
28. Ho, K-W. Risk Factors of Adolescent Pathological Gambling: Permissive Gambling Culture and Individual Factors. *Deviant Behav* **2017**, *38*(5), 533-548.
29. James, R.J.E.; O'Malley, C.; Tunney, R.J. Understanding the psychology of mobile gambling: A behavioural synthesis. *Brit J Psychol* **2017**, *108*(3), 608-625.
30. Kairouz, S.; Fiedler, I.; Monson, E.; Arsenault, N. Exploring the effects of introducing a state monopoly operator to an unregulated online gambling market. *J Gambl Iss* **2017**, *37*, 136-148.

31. Khazaal, Y.; Chatton, A.; Achab, S.; Monney, G.; Thorens, G.; Dufour, M.; Zullino, D.; Rothen, S. Internet gamblers differ on social variables: A latent class analysis. *J Gambl Stud* **2017**, *33*(3), 881-897.
32. Kristiansen, S.; Trabjerg, C.M. Legal gambling availability and youth gambling behaviour: A qualitative longitudinal study. *Int J Soc Welf* **2017**, *26*(3), 218-229.
33. Montes, K.S.; Weatherly, J.N. Differences in the gambling behavior of online and non-online student gamblers in a controlled laboratory environment. *J Gambl Stud* **2017**, *33*(1), 85-97.
34. Mulkeen, J.; Abdou, H.A.; Parke, J. A three-stage analysis of motivational and behavioural factors in UK internet gambling. *Pers Indiv Differ* **2017**, *107*, 114-125.
35. Papineau, E.; Lacroix, G.; Sevigny, S.; Biron, J.; Corneau-Tremblay, N.; Lemetayer, F. Assessing the differential impacts of online, mixed, and offline gambling. *Int Gambl Stud* **2018**, *18*(1), 69-91.
36. Petry, N.M.; Gonzalez-Ibanez, A. Internet gambling in problem gambling college students. *J Gambl Stud* **2015**, *31*(2), 397-408.
37. Redondo, I. Assessing the risks associated with online lottery and casino gambling: A comparative analysis of players' individual characteristics and types of gambling. *International J Ment Health Addict* **2015**, *13*(5), 584-596.
38. Remond, J-J.; Romo, L. Analysis of gambling in the media related to screens: Immersion as a predictor of excessive use? *Int J Environ Res Public Health* **2018**, *15*(1), 58.
39. Sigerson, L.; Li, A.Y.L.; Cheung, M.W.L.; Cheng, C. Examining common information technology addictions and their relationships with non-technology-related addictions. *Computers Human Behav* **2017**, *75*, 520-526.
40. Stead, M.; Dobbie, F.; Angus, K.; Purves, R.I.; Reith, G.; Macdonald, L. The online bingo boom in the UK: A qualitative examination of its appeal. *PloS One* **2016**, *11*(5), e0154763.
41. Talberg, O.N. Can we expect more students dropping out of education to play poker or has online poker become too challenging? *J Gambl Iss* **2017**, *37*, 59-86.
42. Wijesingha, R.; Leatherdale, S.T.; Turner, N.E.; Elton-Marshall, T. Factors associated with adolescent online and land-based gambling in Canada. *Addict Res Theory* **2017**, *25*(6), 525-532.
43. Yuan, J. Examining the gambling behaviors of Chinese online lottery gamblers: are they rational? *J Gambl Stud* **2015**, *31*(2), 573-584.
44. Zhang, M.; Yang, Y.; Guo, S.; Cheok, C.; Kim Eng, W.; Kandasami, G. Online gambling among treatment-seeking patients in Singapore: A cross-sectional study. *Int J Environ Res Public Health* **2018**, *15*(4), 832.

Table S6. Summary of articles on video gaming and gambling.

Author(s) & year/ Study location	Article type	Aim	Key findings	Gaps identified by the authors
Abarbanel & Rahman (2015)[1]<br><br>USA	Cross-sectional research	To examine differences in demographics and gambling behaviours for different frequencies of social casino participation among real money online gamblers	Female real money gamblers, and those who did not complete high school, participated in social casino games with significantly higher frequency. Players who participated frequently in social casino games were likely to spend more time in real money online gambling	**Method gap [Use of improved outcome measures]**<br>Future research should investigate a dataset of social casino players and look at their real money gambling behaviour
Armstrong et al. (2018)[2]<br><br>-	Narrative review	To review the literature exploring simulated gambling products and how consumption may promote monetary gambling, as well as fostering pro-gambling attitudes among youth and adolescents	Youth are highly exposed to simulated gambling games.<br>Those who engage with these products are also more likely to be prone to monetary gambling and gambling problems. Virtual currency, in-game events and gambling themed content are also likely to promote<br>biases about gambling or desensitise consumers to monetary losses	**Knowledge gap [More research in general]**<br>Further research is needed on determining the causal pathway between simulated gambling involvement and monetary gambling in order to identify and manage any risk associated simulated gambling participation
Derevensky & Gainsbury (2016)[3]<br><br>-	Narrative review	To review the literature on social casino gaming and adolescents to examine whether we should be concerned over its widespread use and whether such social games should be regulated	Social gaming site are particularly attracted to youth and the authors recommend that minors should not be targeted, that the graphics should refrain from incorporating childlike characters, that underage minors be prohibited from playing on such sites, and that warnings should be included stating that winning on these sites may not mean that individuals will win when gambling for real money versus virtual money. Responsible gambling frameworks and strategies used in many online gambling sites should be included.	**Method gaps [Longitudinal research]**<br>Longitudinal research is needed to address issues of causality and convergence<br><br>**Public health/practical knowledge gap [Responsible gambling]**<br>Future research should also explore the extent to which the popularity and use of gambling-style games can be developed to have positive educational benefits and facilitate responsible attitudes toward gambling
Drummond & Sauer (2018)[4]<br><br>-	Discussion paper	Presents an analysis and discussion of the characteristics of video game loot boxes in 22 games	Loot boxes have important structural and psychological similarities with gambling	**Knowledge gap [Research on specific factors: Gambling outcomes]**<br>Research is needed to determine whether games that meet the psychological definition of gambling but do not include ability to cash out winnings, and those that do not meet all

				criteria of gambling but involve the exchange of money for randomised rewards, result in adverse short or long-term consequences
Dussault et al. (2017)[5]<br><br>Canada	Longitudinal research	To assess the potential transition from playing with simulated gambling games to gambling with real money in adolescents	At follow-up, 28.8% of the participants had gambled for the first time with real money. Results also revealed that the predictive association between simulated gambling and gambling with real money only held for adolescents who transitioned from simulated poker to poker with real money	**Knowledge gap [More research in general]**<br>Further research is needed to assess the mechanisms at work to understand the impact of simulated gambling<br><br>**Method gap [Use of improved outcome measures]**<br>Measure of simulated gambling can be more refined by taking into account the type of environment involved (e.g. practice/demo games provided by gambling industries)
Floros (2018)[6]<br><br>-	Narrative review	To review the literature on gambling disorder in adolescents	There has been little progress in the standardization of prevention and treatment efforts. There is a large body of research available that clearly demonstrates the extent of the issue with problem gambling and its negative impact on those affected; however, little attention has been devoted to the technological evolution leading to a host of unregulated gambling opportunities, either with real or simulated funds, or with other notions of value to the gambler who may not even identify himself as being a gambler	**Method gaps [Longitudinal research]**<br>Longitudinal studies of those adolescents that are active with simulated gambling and other forms of gambling-like behaviours are needed
Forrest et al. (2016)[7]<br><br>Australia	Cross-sectional research	To examine concurrent video gaming and gambling habits	Gambling involvement was found to be a generally unpopular activity among regular video game players. No significant association was found between frequency of video game play and frequency of gambling	**Knowledge gap [Research on specific factors: Gambling promotion]**<br>Future research should examine the impact of gambling promotion exposures on video game players, and how they avoid gambling engagement
Frahn, Delfabbro & King (2015)[8]<br><br>Australia	Randomised controlled trial	To examine the influence of gambling practice modes on gambling persistence and risk taking, and perceptions of control over gambling outcomes	Players exposed to inflated or 'profit' demonstration modes placed significantly higher bets in the real-play mode as compared to the other groups. However, the groups did not differ in relation to how long they persisted in the real-play mode. Also, pop-up messages had no significant effect on monetary gambling behaviour	**Knowledge gap [Sub-populations]**<br>Further research is needed on the potential risks of simulated gambling activities for vulnerable segments of the gambling population
Gainsbury, Hing, Delfabbro,	Qualitative research	To explore the interrelationships	Respondents reported frequent exposure to promotions for social casino games and that	**Method gap [More representative samples/other sampling gaps]**

Dewar & King (2015)[9]<br><br>Australia		between social casino games, gambling, and problem gambling	being connected to a social network of players was a significant factor in determining their engagement in these activities. However, involvement in social casino games did not appear to affect the likelihood of gambling or the risk of problem gambling. Some problem gamblers did report, however, that these games could sometimes trigger a desire to engage in gambling. Social casino games were commonly perceived as a safe activity that may act as a substitution for gambling	Insights revealed in this study require further investigation using a larger and more diverse sample to enable greater generalisability of the findings<br><br>**Knowledge gap [Research on specific factors: Gambling outcomes]**<br>Future research is needed on the possibility of social casino games as a potentially safe substitution for gambling<br><br>**Method gap [Qualitative research]**<br>Further qualitative research is needed to explore people's lived experiences of social casino gambling;<br>Sociological analyses could focus on, for example, social relationships, interactions, networks, and communities
Hayer et al. (2018)[10]<br><br>Germany	Longitudinal research	To examine if simulated gambling activities predicted gambling with real money during adolescents	12% of the adolescents belonged to the subgroup of 'onset gamblers' and first reported experience with monetary gambling at the second stage of surveying. Logistic regression analysis demonstrated that this migration process was fostered by 1) participation from home in simulated gambling on social networks, and 2) significant exposure to advertising	**Knowledge gap [More research in general]**<br>**Method gap [Use of improved outcome measures]**<br>Future research should consider the complex notion of the gateway effect including (simulated/real) gambling type, mode of access and a more precise definition of migration
Hollingshead et al. (2016)[11]<br><br>Canada	Cross-sectional research	To test the idea that motivation to play social casino games would predict changes in self-reported gambling behaviour among disordered gamblers	Results showed that disordered gamblers who were motivated to play social casino games for the social connection it provided or for skill building, reported an increase in their gambling. Conversely, playing in order to cope with negative life events or for excitement was not predictive of gambling. However, gamblers who reported playing social casino games to reduce cravings to gamble reported an overall decrease in gambling	**Knowledge gap [Research on specific factors: Gambling outcomes]**<br>Further research is needed to explore the potential that social casino games are not solely bad for all disordered gamblers<br><br>**Method gap [Use of improved outcome measures]**<br>A more objective measure of changes in the amount of time and money spent gambling subsequent to playing social casino games would provide a more accurate representation of how social casino gaming influences gambling
Kim et al. (2015)[12]<br><br>Canada	Longitudinal research	To examine the extent to which online social casino gamers migrate to online gambling and to identify the potential predictors of such migration	At follow-up, approximately 26% of online social casino* gamers reported having migrated to online gambling. Importantly, engagement in micro-transactions was the only unique predictor of migration from social casino gaming to online gambling	**Knowledge gap [More research in general]**<br>Further research is needed on the relationship between social casino games and online gambling

Kim et al. (2016)[13]<br><br>Canada	Qualitative research	To explore the potential link between social casino games and online gambling	While many young adults felt immune to effects of social casino games, there was a general consensus that social casino games might facilitate the transition to online gambling among younger teenagers (aged 12-14), due to the ease of accessibility and early exposure	**Knowledge gap [More research in general]**<br>More studies are needed to further examine the link between social casino games and online gambling
Kim et al. (2017)[14]<br><br>Canada	Qualitative research	To explore the motivations to transition from social casino games to online gambling in young adults	Participants noted the role of peer influence as well as incentives (e.g., sign up bonuses) as important factors that motivated them to start engaging in online gambling. Participants also noted a link between social casino games and online gambling. Specifically, several young adults reported migrating to online gambling within a relatively short period after engaging with social casino games. Potential mechanisms that may lead to the migration from social casino games to online gambling included the role of advertisements and the inflated pay out rates on these free to play gambling like games	**Knowledge gap [More research in general]**<br>**Knowledge gap [Sub-populations]**<br>Further research is needed in this domain to mitigate the potential migration from simulated gambling to gambling, specifically amongst those most vulnerable
King et al. (2015)[15]<br><br>-	Discussion paper	To review the principles sources of overlap between gaming and gambling activities in terms of interactivity, monetisation, betting and wagering, types of outcomes, structural fidelity, context and centrality of content, and advertising	Gaming is principally defined by its interactivity, skill-based play, and contextual indicators of progression and success. In contrast, gambling is defined by betting and wagering mechanics, predominantly chance-determined outcomes, and monetisation features that involve risk and payout to the player	None identified
King & Delfabbro (2016)[16]<br><br>Australia	Cross-sectional research	To examine the types and extent of perceived parental influences on adolescents' gambling activities, including whether there might exist differences across commercial and	Financial gambling was usually facilitated by a parent, particularly for scratch tickets and sports betting. Simulated gambling activities, such as social casino games, tended to be reported as being unsupervised by parents. Young people's perceptions of parents' measures to limit, restrict or oversee online and electronic activities were not significantly associated with	**Method gap [Qualitative research; Experimental research]**<br>**Knowledge gap [Sub-populations]**<br>Clinical and qualitative studies of adolescents engaged in gambling, including populations from mental health settings, is needed in order to cross-validate survey data on adolescent problem gambling

		simulated types of activities	youth simulated gambling. Their perceptions of parental influences were not significant predictors of problem gambling behaviours	
King & Delfabbro (2016)[17]<br><br>-	Narrative review	1. Review research on the known risks and benefits of early exposure to simulated gambling.<br>2. Examine exposure models of gambling to aid in classifying these risks and benefits.<br>3. Conceptualise identified factors within a comprehensive theoretical account.	The authors present a two-pathway model that conceptualizes the potential risks and benefits of early exposure to a variety of digital simulated gambling activities (e.g., 'free-to-play' online casinos, gambling like video games, and social casino games). The catalyst pathway describes risk factors associated with early exposure to simulated gambling that may increase the risk of problem gambling. The containment pathway describes protective factors that may increase the likelihood of disinterest in gambling, or a tendency to engage in safe and responsible gambling	**Method gap [Use of data mining]**<br>There is a need for studies that use objective player account data to examine uptake, behavioural patterns, and monetary expenditure in adolescent users<br><br>**Knowledge gap [Research on specific factors: Gambling intentions/behaviours/actions]**<br>There is a need to reassess young people's ability to discriminate between various types of digital and online gambling, given that the nature of gambling is changing rapidly within an evolving technological context
Kristiansen et al. (2018)[18]<br><br>Denmark	Qualitative research (longitudinal)	To explore the types of simulated games and gambling platforms used by adolescents, adolescent's experiences, motivations and behaviours vis a`-vis simulated gambling and the potential interrelationships between simulated and monetary forms gambling	Enjoying social interactional effects appeared to be the main reasons young people engage in simulated gambling games. The study documented characteristics of both a catalyst pathway and a containment pathway emphasizing that for some young people simulated gambling may increase the likelihood of involvement in real money gambling while it may decrease it for others	**Knowledge gap [Research on specific factors: Psychological]**<br>More research is needed to explore and clarify the psychological mechanisms at play in various forms of online simulated gambling, which may help understand how players are induced to continue playing or to transition from simulated gambling to monetary gambling forms
Macey & Hamari (2018)[19]<br><br>Finland	Cross-sectional research	To investigate relationships between a range of gambling activities and the consumption of video games in general, and the newly emergent phenomenon of Esports in particular	Video game addiction was found to be negatively associated with offline gambling, online gambling and problem gambling. Also, video game consumption had only small, positive association with video game-related gambling and problem gambling. Consumption of Esports had small to moderate association with video game-related gambling, online and problem gambling	**Method gap [Use of improved outcome measures]**<br>There is a need for specific measurement tools to be developed rather than using those derived mainly from substance use disorders<br><br>**Knowledge gap [Research on specific factors: Gambling intentions/behaviours/actions]**

				The role and effect of esports, rather than video gaming per se, should be taken into consideration when evaluating the potential to develop problematic gambling behaviours
McBride & Derevensky (2016)[20]<br><br>Canada	Cross-sectional research	To examine commonalities between gambling behaviour and problem gambling among video game players and between video game playing and addicted playing among gamblers	Gamblers, relative to non-gamblers, were more likely to play video games. Video game players were more likely than nonplayers to gamble. Both social and problem gamblers had higher rates of video game playing than did non-gamblers, and addicted gamers had higher rates of gambling than did social and non-gamers	**Knowledge gap [Research on specific factors: Gambling intentions/behaviours/actions; Psychological]**<br>It would be of interest to determine the extent to which individuals distinguish between gambling and video game playing with respect to skill in determining the outcome;<br>Further research into motivations for playing on the basis of gender would help to inform treatment strategies<br><br>**Method gap [More representative samples/other sampling gaps]**<br>Research with young people who are not in school would provide valuable insight into the gambling behaviour of young adults in general
Molde et al. (2018)[21]<br><br>Norway	Longitudinal	To examine if video games were a gateway to gambling	There was a positive relationship between scores on problematic gaming and later scores on problematic gambling, whereas there was no evidence of the reverse relationship	**Knowledge gap [More research in general]**<br>Future research should continue monitoring the possible reciprocal behavioural influences between gambling and video gaming
Sanders & Williams (2018)[22]<br><br>Canada	Cross-sectional research	To examine the relationship between video gaming, gambling and their problematic levels	Most past year video gamers reported gambling (78.5%) and most past year gamblers reported playing video games in the past year (70.7%). Both problem gamers and gamblers had similar demographics, high rates of mental health problems, and impulsivity. Although the risk factors and manifestation of problem gaming and problem gambling were similar, involvement and/or over-involvement in one was not a strong predictor of involvement and/or over-involvement in the other	**Knowledge gap [More research in general]**<br>Further research is needed to understand whether video gaming serves as an entry point or 'getaway' to gambling
Tecihert et al. (2017)[23]<br><br>Germany	Cross-sectional research	To gather an in-depth understanding about the multifacetal market space of online gambling and gaming from a consumer perspective	Products encountering elements of skill, planning, consideration, and achievements over time were perceived differently from those that are more playful and less realistic with immediate outcomes. Consumers did see connections between games and some gambling	**Knowledge gap [More research in general; Sub-populations]**<br>Further research is needed to assess to which extent online gaming can be a gateway to gambling, including among young people and those more vulnerable to the development of gambling problems

			products, which may explain the joint usage of and migration between products	**Knowledge gap [Research on specific factors: Psychological]** Further research is needed to explore how gamblers perceive various gambling activities<br><br>**Public health/practical knowledge gap [Gambling regulation]** Further research is needed to investigate what regulation and policies might be useful to reduce the gateway effect<br><br>**Method gap [Qualitative research]** Further research could include more in-depth qualitative components to verify the results
Wohl et al. (2017)[24]<br><br>-	Narrative review	To discuss the current state of theory and empirical research on the link between social casino gaming and gambling	The transition from social casino gaming to gambling is neither inevitable nor unidirectional. We also outlined a path model of social casino gaming to help delineate the varied effects that it may have on gambling. However, the model will likely require amendments as the literature on social casino gaming continues to grow	**Knowledge gap [More research in general]** More research is needed before strong claims on the good, the bad, or the ugly effects of social casino gaming can be made

* Note: 'Social casino' is the term used to refer to online simulated casino games.

Video gaming and gambling references

1. Abarbanel, B.; Rahman, A. eCommerce market convergence in action: Social casinos and real money gambling. *UNLV Gaming Res Rev J* **2015**, *19*(1), 51-62.
2. Armstrong, T.; Rockloff, M.; Browne, M.; Li, E. An exploration of how simulated gambling games may promote gambling with money. *J Gambl Stud* **2018**, *24*, 1165-1184.
3. Derevensky, J.L.; Gainsbury, S.M. Social casino gaming and adolescents: Should we be concerned and is regulation in sight? *Int J Law Psychiat* **2016**, *44*, 1-6.
4. Drummond, A.; Sauer, J. Video game loot boxes are psychologically akin to gambling. *Nat Human Behav* **2018**, *2*, 530-532.
5. Dussault, F.; Brunelle, N.; Kairouz, S.; Rousseau, M.; Leclerc, D.; Tremblay, J.; Cousineau, M-M.; Dufour, M. Transition from playing with simulated gambling games to gambling with real money: A longitudinal study in adolescence. *Int Gamb Stud* **2017**, *17*(3), 386-400.
6. Floros, G.D. Gambling disorder in adolescents: Prevalence, new developments, and treatment challenges. *Adolesc Health Med Ther* **2018**, *9*, 43-51.
7. Forrest, C.J.; King, D.L.; Delfabbro, P.H. The gambling preferences and behaviors of a community sample of Australian regular video game players. *J Gamb Stud* **2016**, *32*(2), 409-420.
8. Frahn, T.; Delfabbro, P.; King, D.L. Exposure to free-play modes in simulated online gaming increases risk-taking in monetary gambling. *J Gambl Stud* **2015**, *31*(4), 1531-1543.
9. Gainsbury, S.M.; Hing, N.; Delfabbro, P.; Dewar, G.; King, D.L. An exploratory study of interrelationships between social casino gaming, gambling, and problem gambling. *Int J Ment Health Ad* **2015**, *13*(1), 136-153.
10. Hayer, T.; Kalke, J.; Meyer, G.; Brosowski, T. Do simulated gambling activities predict gambling with real money during adolescence? Empirical findings from a longitudinal study. *J Gambl Stud* **2018**, *34*, 929-947.

11. Hollingshead, S.J.; Kim, H.S.; Wohl, M.J.; Derevensky, J.L. The social casino gaming-gambling link: Motivation for playing social casino games determines whether self-reported gambling increases or decreases among disordered gamblers. *J Gambl Stud* **2016**, *33*, 52-67.
12. Kim, H.S.; Wohl, M.J.; Salmon, M.M.; Gupta, R.; Derevensky, J. Do social casino gamers migrate to online gambling? An assessment of migration rate and potential predictors. *J Gambl Stud* **2015**, *31*(4), 1819-1831.
13. Kim, H.S.; Wohl, M.J.A.; Gupta, R.; Derevensky, J. From the mouths of social media users: A focus group study exploring the social casino gaming-online gambling link. *J Behav Addict* **2016**, *5*(1), 115-121.
14. Kim, H,S.; Wohl, M.J.; Gupta, R.; Derevensky, J.L. Why do young adults gamble online? A qualitative study of motivations to transition from social casino games to online gambling. *Asian J Gambl Issues Public Health* **2017**, *7*(1), 1-11.
15. King, D.L.; Gainsbury, S.M.; Delfabbro, P.H.; Hing, N.; Abarbanel, B. Distinguishing between gaming and gambling activities in addiction research. *J Behav Addict* **2015**, *4*(4), 215-220.
16. King, D.L.; Delfabbro, P.H. Adolescents' perceptions of parental influences on commercial and simulated gambling activities. *International Gambling Studies* **2016**, *16*(3): 424-441.
17. King, D.L.; Delfabbro, P.H. Early exposure to digital simulated gambling: A review and conceptual model. *Computers Human Behav* **2016**, *55*, 198-206.
18. Kristiansen, S.; Trabjerg, M.C.; Lauth, N.R.; Malling, A. Playing for fun or gambling for money: a qualitative longitudinal study of digitally simulated gambling among young Danes. *Young Consumers* **2018**, *19*(3), 251-266.
19. Macey, J.; Hamari, J. Investigating relationships between video gaming, spectating esports, and gambling. *Computers Human Behav* **2018**, *80*, 344-353.
20. McBride, J. Gambling and video game playing among youth. *J Gambl Iss* **2016**, *34*, 156-178.
21. Molde, H.; Holmøy, B.; Merkesdal, A.G.; Torsheim, T.; Mentzoni, R.A.; Hanns, D.; Sagoe, D.; Pallesen. S. Are video games a gateway to gambling? A longitudinal study based on a representative Norwegian sample. *J Gambl Stud* **2018**, *35*, 545-557.
22. Sanders, J.; Williams, R. The relationship between video gaming, gambling, and problematic levels of video gaming and gambling. *J Gamble Stud* **2018**, *35*, 559-569.
23. Teichert, T.; Gainsbury, S.M.; Muhlbach, C. Positioning of online gambling and gaming products from a consumer perspective: A blurring of perceived boundaries. *Computers in Human Behav* **2017**, *75*, 757-765.
24. Wohl, M.J.; Salmon, M.M.; Hollingshead, S.J.; Kim, H.S. An examination of the relationship between social casino gaming and gambling: The bad, the ugly, and the good. *J Gambl Iss* **2017**, *35*, 1-23.

Table S7. Summary of articles on the expansion of the sports betting market.

Author(s) & Year/Study Location	Article Type	Aim	Key Findings	Gaps Identified by the Authors
Deans et al. (2016)[1]<br><br>Australia	Mixed-methods research (content analysis)	To explore the written, verbal and visual symbolism used within a sample of 85 sports wagering advertisements	Ten major appeal strategies emerged targeting mostly young males; symbols relating to sports fan rituals and behaviours, and mateship were the most common strategies used within advertisements	**Knowledge gap [More research in general]**<br>**Public health/practical knowledge gap [Gambling regulation]**<br>Future research should try to better understand the impact of marketing on the normalisation of sports wagering on young males, and explore ways to prevent related gambling harm
Deans, Thomas, Daube & Derevensky (2017)[2]<br><br>Australia	Qualitative research	Explored the way in which peer group behaviours influenced attitudes towards, and the consumption of, gambling products	Young men perceived that sports wagering was a 'normal' and socially accepted activity, and a natural 'add on' to sports. There were clear indicators that sports wagering was becoming embedded within existing peer-based sporting rituals, with the emergence of gambling clubs, and online forums. The shaping of gambling/sport discussions created a sense of identity and a point of conversation for peers. Some participants spoke of the social pressure to gamble to 'fit in' with their friends	**Knowledge gap [Research on specific factors: Social factors]**<br>Further research should explore and compare peer norms and influences related to other forms of gambling
Deans, Thomas, Derevensky & Daube (2017)[3]<br><br>Australia	Qualitative research	To explore the attitudes and opinions of men who gambled on sports regarding sports betting marketing, the embedding of marketing within sports and other non-gambling community environments, and the implications this had for the normalisation of betting	Most of the environments in which participants reported seeing or hearing betting advertisements were not in environments specifically designed for betting. Participants described that the saturation of marketing for betting products, including through sports-based commentary and sports programming, normalised betting. Participants described that the inducements offered by the industry were effective marketing strategies in getting themselves and other young men to bet on sports. Inducements were also linked with feelings of greater control over betting outcomes and stimulated some individuals to sign up with more than one betting provider	**Knowledge gap [Sub-populations]**<br>Further research should seek to explore the influence of marketing on the betting attitudes and behaviours of other population subgroups, including female sports fans, and younger populations, such as children and adolescents

Dwyer, Shapiro & Drayer (2018)[4]<br><br>USA	Cross-sectional research	To examine problem gambling severity in conjunction with daily fantasy football participant motives, perceptions, and consumption behaviour	The results suggest daily fantasy football participants behave similarly with participants in other forms of gambling activities	**Knowledge gap [Sub-populations]**<br>Further research is needed to delineate attitudes and consumptive patters based for distinct problem gambling behaviour groups;<br><br>**Knowledge gap [Research on specific factors: Gambling promotion]**<br>More research is need in the area of advertising in the context of daily fantasy football<br><br>**Knowledge gap [Applicability to other gambling types]**<br>Future studies should look to examine professional baseball, basketball, and hockey, as these competitions are truly daily because of the rolling schedules
Dwyer & Weiner (2018)[5]<br><br>USA	Cross-sectional research	To explore the mediating impact of emotions between motives of fantasy participation (i.e., causality orientations) and consumption behaviour between daily and traditional-only fantasy football	The results suggest the differences between the activities are not extreme. However, differences were found in which causality orientations influenced enjoyment and which emotion mediated the relationship between perceived skill and consumption	**Knowledge gap [Research on specific factors: Psychological]**<br>Additional research is suggested to further understand both the perceptions of skill and chance among chasing behaviour, but also the actual skill and chance aspects required in participation;<br>More research within daily fantasy football participation as it relates to anxiety, skill and chance, and engagement is suggested<br><br>**Knowledge gap [More research in general]**<br>More research comparing the two forms of fantasy participation is needed
Gassmann et al. (2017)[6]<br><br>Germany	Cross-sectional research	To explore who bets on sports?	The typical sports-bettor is 32 years old and male, has a low household income, is highly interested in sports, and is willing to take risks	**Knowledge gap [More research in general]**<br>Further research is needed to explore whether participation in sports bets in linked to income<br>Further research is needed to explore the link between the threat of corruption in sports and sports betting;<br>The question of 'who bets on sports?' should be examined in future research
Hing et al. (2016)[7]<br><br>Australia	Cross-sectional research	To identify demographic, behavioural and normative risk factors for gambling problems amongst sports bettors	Vulnerable sports bettors for higher risk gambling are those who are young, male, single, educated, and employed full-time or a full-time student. Risk of problem gambling was also found to increase with greater frequency and	**Method gap [More representative samples/other sampling gaps; Longitudinal]**<br>**Knowledge gap [Replication in other locations]**

			expenditure on sports betting, greater diversity of gambling involvement, and with more impulsive responses to betting opportunities, including in-play live action betting. Normative influences from media advertising and from significant others were also associated with greater problem gambling risk	Further research is needed with representative samples, in other jurisdictions and using prospective designs to confirm the current results<br><br>**Knowledge gap [Research on specific factors: Psychological; Social]**<br>Future studies could also examine other types of risk factors, including psychological, social and cultural variables<br><br>**Knowledge gap [Other/related technologies/trends]**<br>Research into the role of sports betting inducements, such as sign-up bonuses, 'free' bets, money-back guarantees and other prolific offers, would also help to identify their influence on individuals' commencement, continuation and intensification of sports betting
Hing, Russell, Lamont & Vitartas (2017)[8]<br><br>Australia	Cross-sectional research	Examined whether responses to for online sports betting during televised sports broadcasts varied with problem gambling severity	Young male internet sports bettors were more vulnerable to these promotions, particularly if they held positive attitudes towards the sponsors	**Method gaps [Longitudinal research]**<br>Further prospective studies should aim to untangle causal relationships between exposure to promotions, related attitudes and problem gambling
Li et al. (2015)[9]<br><br>China	Cross-sectional research	To classify and profile sports lottery gamblers	5 distinctive clusters of sports gamblers were identified: casual, escalated, at-risk, compulsive and problem players. They all differed in both terms of demographic and behavioural characteristics	**Knowledge gap [More research in general]**<br>More research is needed on this topic to confirm these findings
Lin & Lu (2015)[10]<br><br>Taiwan	Cross-sectional research	To elucidate the association of sports lottery bettors' socio-demographics, personality traits, risk tolerance and behavioural biases	The bettors with neuroticism have lower risk tolerance, and the bettors with greater neuroticism, openness and agreeableness exhibit apparent normative herding behaviours	**Knowledge gap [Research on specific factors: Psychological]**<br>Future studies can explore the motivations of sports lottery bettors for participating in the underground economy (i.e. illegal betting) and compare the economic scale with the legitimate betting market to reveal other key factors that influence the decision-making of bettors
Lopez-Gonzalez, Guerrero-Sole & Griffiths (2018)[11]	Mixed-methods research	Examined a sample of British and Spanish sports betting television adverts to understand how	Results showed a male-dominant betting representation with no interaction between women. Typically, bettors were depicted surrounded by people but isolated in their	**Knowledge gap [Research on specific factors: Gambling promotion]**<br>Further research is needed on in-play betting in advertising

UK & Spain		bettors and betting were being represented	betting, emphasising the individual consumption practice that mobile betting promotes. Results indicated that betting while watching sport in betting adverts is associated with emotionally charged situations such as celebrations and/or alcohol drinking. Bettors were typically depicted staking small amounts of money with large potential returns, implying high risk bets	
Lopez-Gonzalez, Estevez, Jiminez-Murcia & Griffiths (2018)[12]<br><br>UK & Spain	Mixed-methods research	Examined the representation of alcohol drinking and low nutritional value food eating in sports betting advertising	The results suggested that betting advertising aligned drinking alcohol with sports culture and significantly associated emotionally charged sporting situations such as watching live games or celebrating goals with alcohol. Additionally, alcohol drinking is more frequent in betting adverts with a higher number of characters, linking friendship bonding and alcohol drinking (especially beer) in the context of sports gambling	**Knowledge gap [Research on specific factors: Gambling intentions/behaviours/actions]**<br>Future research will need to evaluate whether new forms of advertising are having an impact on the co-occurrence of these three behaviours
Lopez-Gonzalez, Estevez & Griffiths (2018)[13]<br><br>UK	Qualitative research	Grounded theory study of a British sample of sports betting advertisements	Individual themes aligned in a single core narrative, constructing a dual persuasive strategy of sports betting advertising: i) to reduce the perceived risk involved in betting while ii) enhancing the perceived control of bettors	**Knowledge gap [Research on specific factors: Psychological]**<br>Researchers should further examine the development of latent narratives, such as those of control, to ascertain whether they have a negative impact on the conceptualisation and shaping of sports betting behaviour in bettors' minds
Marchica & Derevensky (2016)[14]<br><br>USA	Longitudinal research	Explored the prevalence and growth in fantasy sports among student-athletes	Overall, a steady increase in fantasy sports participation (for money or fun) in college was found. Additionally, approximately half of college student-athletes who qualified as at risk or having gambling problems were found to have participated in fantasy sports wagering	**Knowledge gap [Research on specific factors: Psychological]**<br>Further research looking into student-athlete perceptions would be necessary in order to provide additional information on their motives
Marchica et al. (2017)[15]<br><br>USA	Cross-sectional research	Examined the relationship between regular participation (more than once a month) in sport-relevant gambling activities among adolescents and those identified as being	Regular involvement in sports betting, fantasy sports betting, and daily fantasy sports betting among adolescents was associated with a higher risk of gambling problems. Further, although males participate more frequently in these activities, females who participate have a stronger likelihood of being at-risk. Students aged 16–19 years old are at a higher risk for developing a gambling problem compared to	**Method gap [More representative samples/other sampling gaps]**<br>Future studies should investigate the participation rates and impact of fantasy sports (both daily and league based) among adolescents on a larger scale in order to identify how this growing activity may affect problem gambling behaviours

		at-risk for a gambling problem	younger adolescents when regularly engaging in sports-related gambling. Moreover, regularly participating in daily fantasy sports is the strongest predictor of at-risk gambling behaviour in 13–15 year old students. A hierarchical logistic regression supports that controlling for gender and age, all forms of sport-relevant gambling activities are significant predictors of at-risk gambling	
Martin et al. (2016)[16]<br><br>USA	Cross-sectional research	To examine gambling behaviour (past-year gambling, gambling-related problems, and fantasy sport gambling) among a sample of college students at a private religiously affiliated university in the Southwest US	Compared to females, males observed higher rates of past year gambling, fantasy sports participation, fantasy sports gambling, and gambling-related problems. Among males, we found that club/intramural/recreational athletes observed the highest rates of past-year gambling and fantasy sports participation; Division 1 athletes observed higher rates than non-athletes	None
Newall (2017)[17]<br><br>UK	Comparative study with controls	Soccer fans took part across five experiments (designed based on content analysis of advertisements) to examine the behavioural complexity of British gambling advertising	A content analysis of high-impact televised soccer adverts showed that most advertised gambles were for complex events. Soccer fans rarely formed rational probability judgments for the complex events dominating gambling advertising but were much better at estimating simple events. British gambling advertising is concentrated on the complex products that mislead consumers the most	**Knowledge gap [Applicability to other populations; Applicability to other gambling types; Replication in other locations]**<br>Further research is needed to explore the prevalence of similar gambling advertising over a wide variety of media;<br>Research should investigate similar gambling advertising across other sports and in other countries<br><br>**Knowledge gap [More research in general]**<br>Further research is needed in this area given that results may be an artefact of the experimental situation
Nower et al. (2018)[18]<br><br>USA	Cross-sectional research	To investigate gambling, addiction and mental health problems among daily fantasy sports players	Overall, a higher number of gambling activities, high frequency gambling, male gender and reports of suicidal thoughts in the past year were most predictive of daily fantasy sports players. Being Hispanic and/or single also doubled the odds of daily fantasy sports play	**Knowledge gap [Research on specific factors: Psychological]**<br>Future research should examine the motivations and possible ecological sub-types of daily fantasy sports players and the nature and course of daily fantasy sports play, particularly in relation to gambling behaviour and the development of gambling and other problems

Pitt et al. (2016)[19]<br><br>Australia	Mixed methods research	To investigate how children and adults recall the content and promotional channels for sports wagering marketing	Children recruited from NRL and AFL sites were significantly more likely to have recalled having ever seen a promotion for sports wagering as compared to children from Soccer sites. Children and adults identified seeing sports wagering promotions in similar environments, most commonly on television, and at stadiums. Three-quarters of children and the majority of adults perceived that sports wagering was becoming a normal part of sport	**Knowledge gap [Research on specific factors: Psychological; Gambling intentions/behaviours/actions]**<br>Further research is needed on children's gambling intentions; Further research is needed to understand how a range of promotions may influence children's gambling risk/benefit perceptions, beliefs and attitudes, and future consumption intentions<br><br>**Knowledge gap [Research on specific factors: Gambling promotion]**<br>Further research should examine whether children's brand awareness is higher for companies that spend the most on marketing their products
Pitt et al. (2017)[20]<br><br>Australia	Qualitative research	To explore children's gambling attitudes and consumption intentions and the range of consumer socialisation factors that may influence these attitudes and behaviours	First, children's perceptions of the popularity of different products were shaped by what they had seen or heard about these products, whether through family activities, the media (and in particular marketing) of gambling products, and/or the alignment of gambling products with sport. Second, children's gambling behaviours were influenced by family members and culturally valued events. Third, many children indicated consumption intentions towards sports betting. This was due to four key factors: (1) the alignment of gambling with culturally valued activities; (2) their perceived knowledge about sport; (3) the marketing and advertising of gambling products (and in particular sports betting); and (4) the influence of friends and family	**Knowledge gap [Research on specific factors: Gambling promotion]**<br>Further research is needed into the impact of newer marketing creatives on young people
Pitt et al. (2017)[21]<br><br>Australia	Qualitative research	To explore children's awareness of sports betting advertising and how this advertising may influence children's attitudes, product	Children recalled in detail sports betting advertisements that they had seen, with humour the most engaging appeal strategy. They were also able to describe other specific appeal strategies and link these strategies to betting brands. Many children described how	**Knowledge gap [Research on specific factors: Gambling intentions/behaviours/actions]**<br>Future research should further investigate the links between specific advertising strategies, their 'likeability', brand loyalty, and children' intentions to gamble

		knowledge and desire to try sports betting	advertisements demonstrated how someone would place a bet, with some children recalling the detailed technical language associated with betting	**Knowledge gap [Sub-populations]** Further comparative studies of children who are fans of sporting codes that have significant amounts of betting advertising and sponsorship, sporting codes with limited betting advertising and sponsorship, and children who are not fans of sports will be important in further identifying potential risk factors for different sub-groups of children
Wann et al. (2015)[23] USA	Cross-sectional research	To examine possible changes in fandom by investigating sports fans' perceptions of the impact of increasing legalised sport wagering on their fan experience, interest in sport, and sport consumption	Expected impacts were small and generally positive and these effects were greatest among groups historically active in sport gambling	**Knowledge gap [Research on specific factors: Gambling outcomes]** Future research should examine the manner and extent to which individuals report that increasing the legalisation of sport wagering would impact their online betting and their consumption of sport in the internet

Sports betting references

1. Deans, E.G.; Thomas, S.L.; Daube, M.; Derevensky, J.; Gordon, R. Creating symbolic cultures of consumption: an analysis of the content of sports wagering advertisements in Australia. *BMC Public Health* **2016**, 16, 208.
2. Deans, E.G.; Thomas, S.L.; Daube, M.M.; Derevensky, J. The role of peer influences on the normalisation of sports wagering: A qualitative study of Australian men. *Addict Res Theory* **2017**, *25*(2), 103-113.
3. Deans, E.G.; Thomas, S.L.; Derevensky, J.; Daube, M. The influence of marketing on the sports betting attitudes and consumption behaviours of young men: Implications for harm reduction and prevention strategies. *Harm Reduct J* **2017**, *14*(1), 5.
4. Dwyer, B.; Shapiro, S.L.; Drayer, J. Daily fantasy football and self-reported problem behavior in the United States. *J Gambl Stud* **2018**, *34*(3), 689-707.
5. Dwyer, B.; Weiner, J. Daily Grind: A comparison of causality orientations, emotions, and fantasy sport participation. *J Gambl Stud* **2018**, *34*(1), 1-20.
6. Gassmann, F.; Emrich, E.; Pierdzioch, C. Who bets on sports? Some further empirical evidence using German data. *Int Rev Sociol Sport* **2017**, *52*(4), 391-410.
7. Hing, N.; Russell, A.M.T.; Vitartas, P.; Lamont, M. Demographic, behavioural and normative risk factors for gambling problems amongst sports bettors. *J Gambl Stud* **2016**, *32*(2), 625-641.
8. Hing, N.; Russell, A.M.T.; Lamont, M.; Vitartas, P. Bet Anywhere, Anytime: An analysis of Internet sports bettors' responses to gambling promotions during sports broadcasts by problem gambling severity. *J Gambl Stud* **2017**, *33*(4), 1051-1065.
9. Li, H.; Mao, L.L.; Zhang, J.J.; Xu, J. Classifying and profiling sports lottery gamblers: A cluster analysis approach. *Soc Behav Pers* **2015**, *43*(8), 1299-1318.
10. Lin, H-W.; Lu, H-F. (2015) Elucidating the association of sports lottery bettors' socio-demographics, personality traits, risk tolerance and behavioural biases. *Pers Indiv Diff* **2015**, *73*, 118-126.
11. Lopez-Gonzalez, H.; Guerrero-Sole, F.; Griffiths, M.D. A content analysis of how 'normal' sports betting behaviour is represented in gambling advertising. *Addict Res Theory* **2018**, *26*(3): 238-247.

12. Lopez-Gonzalez, H.; Estevez, A.; Jimenez-Murcia, S.; Griffiths, M.D. Alcohol drinking and low nutritional value food eating behavior of sports bettors in gambling advertisements. *International J Ment Health Ad* **2018**, *16*(1), 81-89.
13. Lopez-Gonzalez, H.; Estevez, A.; Griffiths, M.D. Controlling the illusion of control: A grounded theory of sports betting advertising in the UK. *Int Gambl Stud* **2018**, *18*(1), 39-55.
14. Marchica, L.; Derevensky, J. Fantasy sports: A growing concern among college student-athletes. *Int J Ment Health Ad* **2016**, *14*(5), 635-645.
15. Marchica, L.; Zhao, Y.; Derevensky, J.; Ivoska, W. Understanding the relationship between sports-relevant gambling and being at-risk for a gambling problem among American Adolescents. *J Gambl Stud* **2017**, *33*(2), 437-448.
16. Martin, R.J.; Nelson, S.E.; Gallucci, A.R. Game On: Past year gambling, gambling-related problems, and fantasy sports gambling among college athletes and non-athletes. *J Gambl Stud* **2016**, *32*(2), 567-579.
17. Newall, P.W. Behavioral complexity of British gambling advertising. *Addict Res Theory* **2017**, *25*(6), 505-511.
18. Nower, L.; Caler, K.R.; Pickering, D.; Blaszczynski, A. Daily Fantasy Sports Players: Gambling, Addiction, and Mental Health Problems. *J Gambl Stud* **2018**, *34*(3), 727-737.
19. Pitt, H.; Thomas, S.L.; Bestman, A.; Stoneham, M.; Daube, M. "It's just everywhere!" Children and parents discuss the marketing of sports wagering in Australia. *Aust NZ J Public Health* **2016**, *40*(5), 480-486.
20. Pitt, H.; Thomas, S.L.; Bestman, A.; Daube, M.; Derevensky, J. Factors that influence children's gambling attitudes and consumption intentions: lessons for gambling harm prevention research, policies and advocacy strategies. *Harm Reduct J* **2017**, *14*(11), 1-12.
21. Pitt, H.; Thomas, S.L.; Bestman, A.; Daube, M.; Derevensky, J. What do children observe and learn from televised sports betting advertisements? A qualitative study among Australian children. *Aust NZ J Public Health* **2017**, *41*(6), 604-610.
22. Wann, D.L.; Zapalac, R.K.; Grieve, F.G.; Partridge, J.A.; Lanter, J.R. An examination of sport fans' perceptions of the impact of the legalization of sport wagering on their fan experience. *UNLV Gaming Res Rev J* **2015**, *19*(2), 21-40.

Table S8. Summary of articles on electronic gaming machines (EGMs).

Author(s) & Year/Study Location	Article Type	Aim	Key Findings	Gaps Identified by the Authors
Adams & Wiles (2017)[1]<br><br>-	Discussion paper	Explored is how the design of gambling venues promotes a form of solitary and anonymous play that promotes entry to the zone and, accordingly, entices players into gambling addictively	By examining a series of common layouts we identify the division of venues into two main areas: one for the main social activities of the venue (the "main hall") and the other a partitioned area (the "annex") in which gambling machines are tightly clumped in ways that discourage social interaction. Other features of the annex that encourage uninterrupted and solitary play include the absence of tables to socialize around, dimmed lighting and entry pathways that minimize scrutiny. The authors argue that these features promote a style of play more oriented towards heavy and problematic gambling	**Knowledge gap [More research in general]**<br>Expand and resource gambling research beyond the narrow focus on the player-machine dyad and towards a recognition of the role the annex in promoting problem gambling
Armstrong et al. (2016)[2]<br><br>Australia	Qualitative research	To conduct an Australian national environmental scan of electronically and mechanically enhanced table-game and community-game products to identify the characteristics of these automated products Australia-wide	The findings suggested that automation provided the potentials for provision of products that intensify gambling engagement with the attendant potential for gambling-related harm	**Knowledge gap [Research on specific factors: Gambling intentions/behaviours/actions]**<br>Future research should focus on which features of automated products are most appealing to gamblers, and how they alter relative popularity and safety of different forms of gambling
Armstrong et al. (2017)[3]<br><br>-	Narrative review	Seeks to illustrate how automation is likely to change the way people engage and experience traditional games based around five prominent modifications: visual and auditory enhancements; illusions of control;	The inclusion of rich graphics, event-dependent sound and game-play information such as statistics, history, betting options and strategic betting are likely to prolong and entice gambling while encouraging more intense betting. Changes to the social environment due to the asocial nature of automated products is also likely to significantly change the gambling experience. Given the increasing prevalence of these	**Knowledge gap [More research in general]**<br>More research is needed to determine the full impact of automation on player behaviours in order to understand the potential risks associated with technological enhancements to traditional games

		cognitive complexity; expedited play; and social customisation	products in the marketplace, it is important to consider the implications of converting traditional products to automated form as technological enhancements have the potential to allow for faster, more intense betting	
Barton et al. (2017)[4]<br><br>-	Systematic review	Describes the behavioural, psychological, and psychobiological effects of near misses and losses disguised as wins (LDWs) in an effort to evaluate their precise influence on the player and to highlight areas requiring further investigation	A total of 51 experimental peer-reviewed studies using human participants were found between 1991 and 2015. Near misses motivate continued play, but have varying effects on the emotional state or betting behaviour of the player. Near miss events were also shown to be associated with elevated skin conductance levels and diffuse activity across the brain, most consistently in areas processing reinforcement and reward	**Knowledge gap [Research on specific factors: Psychological]**<br>Betting and gambling behaviour appear to be the result of a potentially large number of different factors which vary between players, each of which must be better identified and measured in further studies;<br>Further research is needed on the ability for near miss events to produce a response in the player at the physiological level;<br>Future work should investigate the specific role of the insula and the complex interplay between tissue in the insular cortex, striatal, and inferior parietal lobule in processing near miss events;<br>Further research is needed to explore the clarity of the near miss event as aversive and to differentiate the various modes of cognitive processing from the excitement of an LDW event
Browne et al. (2015)[5]<br><br>Australia	Mixed-methods research	A pre-play survey of adult gambler, and live observation plus recording of their EGM play measures were conducted to examine the impact of EGM jackpots on players' behaviour	Primed participants were more likely to select jackpot-oriented EGMs, and primed at-risk gamblers tended to select machines with a higher median jackpot prize amount than others. Neither PGSI nor priming was associated with the rate at which participants switched machines. EGM jackpots were associated with great spend overall, and PGSI score was associated with a greater spend per play. Positive interactions were found between jackpots and PGSI, and PGSI and priming in terms of predicting greater persistence	**Method gap [Use of data mining]**<br>Future research could potentially use large datasets gathered by government-or-corporate-sponsored player behaviour tracking technologies, to uncover both between-participant effects, and within-session dynamics
Donaldson et al. (2016)[6]<br><br>Australia	Randomised Controlled Trial	To examine the effects of hidden jackpot, mystery jackpot or control condition (no jackpot) on gambling intensity	Gambling intensity was greater when the jackpot value was unknown, especially when a winning-symbol combination suggested that a win was possible. There is some evidence to suggest a marginal positive contribution of hidden jackpots to risky playing behaviour	**Knowledge gap [Research on specific factors: Psychological]**<br>Future research on hidden jackpots is needed on the mechanisms for the increase in physiological arousal;<br>Future research is needed focusing on player expectancies

Gainsbury & Blaszczynski (2017)[7]<br><br>Australia	Commentary	To provide an overview of virtual reality gambling, current market offerings and regulation, and its future implications for the field of gambling	The gambling environment is moving toward more interactive formats with electronic gaming machines containing skill-based elements. Virtual reality gambling is a logical extension of this development. Regulators and treatment providers need to understand the potential impact of virtual reality gambling	**Knowledge gap [Research on specific factors: Gambling outcomes]**<br>Further research is needed to understand the likely impact of virtual reality gambling on consumers
Goodwin et al. (2017)[8]<br><br>Australia	Qualitative research	To explore player experiences with both traditional and innovated gambling products	Traditional games were perceived as more social, more enjoyable and less harmful. This was largely attributed to the low social interaction, expedited play and increased potential for consumption that is associated with EGM-type gambling activity	**Method gap [More representative samples/other sampling gaps]**<br>Further quantitative research is suggested to validate these findings in a broader and more representative sample
Landon et al. (2016)[9]<br><br>New Zealand	Qualitative research	To explore EGM characteristics	Two groups of EGM characteristics were identified: winning and betting. Overall, free spin features were identified in all groups as the most attractive characteristics of EGMs. The important characteristics were consistent across different levels of gamblers, with the key behavioural difference being a self-reported 'expertise' and 'strategic' approach to gambling amongst higher-frequency gamblers and problem gamblers in treatment	**Knowledge gap [More research in general]**<br>Characteristics such as free spin features, and other frequent small win-related events coupled with low denomination EGMs with multiple playable lines should be the focus of further research
Li et al. (2016)[10]<br><br>Australia	Randomised Controlled Trial	To examine jackpot's rollover effect and goal-gradient effect in EGM gambling	Three major findings: a) Players placed the largest bets (20.3% higher than the average) on large jackpot EGMs that were represented to be deterministic and non-progressive. b) Large jackpot that were non-deterministic and progressive also promoted high bets, resembling the 'rollover effect' demonstrated in lottery betting. c) Neither the hypothesized goal-gradient effect nor the rollover effect was evident among players betting on small jackpot machines	**Knowledge gap [More research in general]**<br>Similar studies should be conducted to confirm these findings- specially in real settings
MacLaren (2016)[11]<br><br>Canada	Cross-sectional research	To examine video lottery terminals' (VLT) impact on problem and pathological gambling (PPG)	The relative risk of PPG was higher among VLT players than it was for other common forms of gambling (e.g. slots, lottery, horse racing). VLT gambling is also the most expensive gambling habit in Canada	**Knowledge gap [Sub-populations]**<br>Future research should aim to answer the degree to which different types of problem and non-problem gamblers prefer multiple forms of gambling

Murch et al. (2019)[12]<br><br>Canada	Comparative study without controls	To examine the effects of bet size and multi-line play on immersion and respiratory sinus arrhythmia (RSA) during EGM use	Results suggested that multi-line EGMs capture attention across a range of play-styles and that immersion might be effectively amplified by multi-line play	**Knowledge gap [Research on specific factors: Psychological]**<br>Future research is needed in applying eye tracking to monitor attentional allocation during EGM play and relationships with immersion
Quilty et al. (2016)[13]<br><br>Australia	Cross-sectional research	To examine the influence of monetary payout on gambling behaviour	Gambling behaviours increased with monetary payout even across qualitatively different gambling products, and across gamblers with different motivations for gambling, levels of impulsivity, and negative affect	**Public health/practical knowledge gap [Gambling regulation]**<br>The restriction of monetary payouts warrants further research attention as a form of problem gambling prevention
Riva et al. (2015)[14]<br><br>Italy	Comparative study with controls	To test whether anthropomorphizing slot machines would increase gambling	Exposing people to an anthropomorphized description of a slot machine increased gambling behaviour	**Knowledge gap [Research on specific factors: Psychological]**<br>Future research should examine the underlying mechanisms of the link between slot machine anthropomorphisation and gambling behaviour, and also investigate how individual differences can impact this link
Worhunsky & Rogers (2018)[15]<br><br>UK	Comparative study without controls	To estimate the individual rate-of-play preferences and associations with EGM gambling behaviour	Estimated I-ROPs ranged from less than one half second to over seven seconds and were negatively associated with cognitive ability, but not related to problem gambling, impulsiveness, or gambling-related cognitions. Subsequent gambling sessions on EGMs offering individually calibrated faster and slower gaming speeds were associated with greater and reduced risk-related gambling behaviours respectively	**Knowledge gap [Research on specific factors: Psychological]**<br>I-ROPs represent a potentially informative construct for exploring influences of gaming speed on gambling behaviour, and may lend insight into potential risk-related behaviour an individual vulnerability with respect to commercially available EGMs that warrants additional research

Electronic gaming machines references

1. Adams, P.J.; Wiles, J. Gambling machine annexes as enabling spaces for addictive engagement. *Health Place* **2017**, *43*, 1-7.
2. Armstrong, T.; Rockloff, M.; Donaldson, P. Crimping the croupier: Electronic and mechanical automation of table, community and novelty games in Australia. *J Gambl Iss* **2016**, *33*, 103-123.
3. Armstrong, T.; Rockloff, M.; Greer, N.; Donaldson, P. Rise of the machines: A critical review on the behavioural effects of automating traditional gambling games. *J Gambl Stud* **2017**, *33*(3), 735-767.
4. Barton, K.R.; Yazdami, Y.; Ayer, N.; Kalvapalle, S.; Brown, S.; Stapleton, J.; Brown, D.G.; Harrigan, KA. The effect of losses disguised as win and near misses in Electronic Gaming machines; A systematic review. *J Gambl Stud* 2017, *33*, 1241-1260.
5. Browne, M.; Langham, E.; Rockloff, M.J.; Li, E.; Donaldson, P.; Goodwin, B. EGM jackpots and player behaviour: An in-venue shadowing study. *J Gambl Stud* **2015**, *31*(4), 1695-1714.
6. Donaldson, P.; Langham, E.; Rockloff, M.J.; Browne, M. Veiled EGM jackpots: The effects of hidden and mystery jackpots on gambling intensity. *J Gambl Stud* **2016**, *32*(2), 487-498.

7. Gainsbury, S.M.; Blaszczynski, A. Virtual reality gambling: public policy implications for regulation and challenges for consumer protection. *Gaming Law Rev* **2017**, *21*(4), 314-322.
8. Goodwin, B.; Thorne, H.; Langham, E.; Moskovsky, N. Traditional and innovated gambling products: An exploration of player preferences. *Int Gambl Stud* **2017**, *17*(2), 219-235.
9. Landon, J.; du Preez, K.P.; Page, A.; Bellringer, M.; Roberts, A.; Abbott, M. Electronic gaming machine characteristics: it's the little things that count. *Int J Ment Health Ad* **2018**, *16*(2), 251-265.
10. Li, E.; Rockloff, M.J.; Browne, M.; Donaldson, P. Jackpot structural features: Rollover effect and goal-gradient effect in EGM gambling. *J Gambl Stud* **2016**, *32*(2), 707-720.
11. MacLaren, V.V. Video Lottery is the Most Harmful Form of Gambling in Canada. *J Gambl Stud* **2016**, *32*(2), 459-485.
12. Murch, W.S.; Clark, L. Effects of bet size and multi-line play on immersion and respiratory sinus arrhythmia during electronic gaming machine use. *Addict Behav* **2019**, *88*, 67-72.
13. Quilty, L.C.; Lobo, D.S.; Zack, M.; Crewe-Brown, C.; Blaszczynski, A. Hitting the jackpot: the influence of monetary payout on gambling behaviour. *Int Gambl Stud* **2016**, *16*(3), 481-499.
14. Riva, P.; Sacchi, S.; Brambilla, M. Humanizing machines: Anthropomorphization of slot machines increases gambling. *J Exp Psychol Appl* **2015**, *21*(4), 313-325.
15. Worhunsky, P.D.; Rogers, R.D. An initial investigation of individual rate-of-play preferences and associations with EGM gambling behavior. *J Gambl Stud* **2018**, 34, 1067-1083.

Table S9. Summary of articles on advertising and inducements.

Author(s) & Year/Study Location	Article Type	Aim	Key Findings	Gaps Identified by the Authors
Abarbanel et al. (2017)[1]<br><br>Australia	Qualitative research	To conduct a content analysis of social gaming advertisements captured by young adults during their regular Internet use	The advertisements were targeted towards young adults. The content focused on glamorization of gambling, winning, normalization, play for free etc. Notably, 90% of the advertisements did not contain responsible or problem gambling language	**Method gap [Qualitative research]**<br>To conduct phenomenological interviews to explore how young adults receive messages and their effects on their attitudes and behaviour<br><br>**Knowledge gap [More research in general; Replication in other locations]**<br>Repetition of this study is recommended to better capture advertisements during other young adult Internet behaviours, and similar studies in other jurisdictions and other languages where cultural factors may be examined are also recommended;<br>A review of current policies as well as further research is needed to establish stronger understanding of the impacts of gambling-themed advertising content
Baloglu et al. (2017)[2]<br><br>USA	Cross-sectional research	To develop and test a model of casino loyalty	Trust, perceived switching cost, and emotional commitment to the casino are more likely to influence relational or emotional outcomes such as word of mouth and voluntary partnership whereas the loyalty program is more likely to influence transactional outcomes such as repeat visitation and time spent in the casino. The emotional commitment served as a partial mediator in the model	**Knowledge gap [More research in general]**<br>Future research can investigate the moderating effect of loyalty program membership on the relationship between switching costs/trust and customer loyalty
Gainsbury, King et al. (2015)[3]<br><br>Australia	Qualitative research	To explore how gambling operators are using social media to engage with users and promote products, their considerations underpinning these actions, and the extent to which responsible	All were active on social media. The platforms were used to maintain customer base and attract new customers, Gambling-related content was usually balanced against non-gambling content. Few operators provided specific responsible gambling messages	**Knowledge gap [More research in general]**<br>Research is needed to explore the use of social media by gambling operators further; for example, by investigating the shift in marketing efforts towards social media and the impact of this marketing on consumers

		gambling practices are included		
Gainsbury, Delfabbro, King & Hing (2016)[4]<br><br>Australia	Qualitative research	Investigated how social media is used by gambling operators to promote gambling activities including an analysis of the latent messages that are conveyed	Facebook and Twitter were the dominant platforms used, most commonly by casinos and online wagering operators. A key finding was that online gambling operators included gambling content with both related news and unrelated content, as way of normalising gambling within a broader social context. Also, most operators did not incorporate RG messages	**Knowledge gap [Research on specific factors: Gambling outcomes]**<br>Further research could investigate consumer engagement with gambling operators on social media, how it impacted consumers' gambling behaviour
Gainsbury, King, Russell, Delfabbro, Derevensky & Hing (2016)[5]<br><br>Australia	Cross-sectional research	To investigate the impact of social media gambling marketing on moderate-risk and problem gamblers	Moderate-risk and problem gamblers were more likely to be impacted by social media promotions, and these might play a role in exacerbating disordered gambling	**Method gap [Use of improved outcome measures]**<br>Future research should verify these self-reported findings with behavioural data
Hing, Sproston et al. (2017)[6]<br><br>Australia	Exploratory research	To document the range and structural features of sports and race betting inducements, and analyse their alignment with the harm minimisation and consumer protection goals of responsible gambling	223 separate inducements were located that were categorised into 15 generic types, all offering financial incentives to purchase. These comprised: sign-up offers, refer-a-friend offers, matching stakes/deposits, winnings paid for 'close calls', bonus or better odds etc. Only few contained responsible gambling messages; play through conditions of bonus bets were particularly difficult to interpret and failed basic requirements for informed choice	**Knowledge gap [Research on specific factors: Gambling intentions/behaviours/actions; Gambling outcomes]**<br>Empirical research is urgently needed to determine the actual influence of different type of inducements on betting attitudes and intentions, on the commencement, continuation and intensification of betting, on different aspects of problem gambling and on consumer protection
Hing, Russell et al. (2018)[7]<br><br>Australia	Cross-sectional research	To examine whether the uptake of wagering inducements predicted impulse betting on sport	More frequent users of wagering inducements had a greater tendency to place impulse in-play bets, which were also predicted by problem gambling, higher buying impulsiveness, higher frequency of watching sports, younger age and higher education status. Sport bettors with a greater tendency to place impulse bets before match commencement also tended to have higher buying impulsiveness and younger, but used inducement less frequently and tended to be	**Method gap [Experimental research]**<br>Additional research employing different methodologies are required to identify casual effects between variables, and to clarify the direction of associations found in this study

			females, less-educated and non-problem, moderate risk or problem gamblers	
O'Loughlin & Blaszczynski (2018)[8]<br><br>Australia	Comparative study without controls	Examined the differential effects of traditional print media (newspapers), and gambling operator and peer postings on Facebook on gambling attitudes and intentions to gamble in a sample of 120 male first-year university students	Gambling attitude and intentions did not differ between averaged peer and gambling operator Facebook postings compared to traditional media. However, gambling advertisements appeared to influence gambling attitudes and medium-term gambling intentions when posted by a gambling operator compared to a peer on Facebook. Gambling advertisements in traditional media and social media are equivalent in their effects on gambling attitudes and intentions	**Public health/practical knowledge gap [Gambling regulation]**<br>There is a need to further explore the implications for regulating social media advertisements
Prentice & Wong (2015)[9]<br><br>China	Cross-sectional research	Examines the mediating role of gambling behaviours in the relationship between commonly practiced customer acquisition and retention strategies in casinos and problem gambling or customer loyalty	Results from testing the hypotheses in the second survey confirmed some proposed relationships and failed to support others. There was a significant effect of 'Fengshui' on problem gambling. Loyalty program had direct and indirect effects on gambling	**Method gap [More representative samples/other sampling gaps]**<br>A larger sample would enable more powerful statistical analyses<br><br>**Knowledge gap [Research on specific factors: Gambling promotion]**<br>More customer acquisition and retention strategies could be identified for comprehensive analyses
Quigno & Zhang (2016)[10]<br><br>USA	Comparative study with controls	Examined the combined effect of number of tiers and gender on customers' attitudes and intention to join a loyalty rewards program	Male customers exhibited more positive attitudes and higher level of intention to join a program that has four tiers (vs two tiers). Conversely, female customers' attitudes and joining intentions were stronger when facing a loyalty rewards program containing two tiers (vs. four tiers). The underlying psychological mechanism was individuals' need for status	None identified
Yoo & Singh (2016)[11]<br><br>USA	Cross-sectional research	To examine the effectiveness of a loyalty program on members' behavioural usage level from different tiering strategies	Study results did not support the research hypothesis that card tiers have a significant effect on behavioural loyalty, indicating that purchase behaviour factors are not significantly related to card tiers	**Knowledge gap [Sub-populations]**<br>Replication of the study is recommended with different random samples drawn from diverse segments of casinos and considering patrons who move tier levels<br><br>**Knowledge gap [Research on specific factors: Psychological]**

			Future studies would benefit by expanding the scope to include information pertaining to attitudinal loyalty

Advertising and inducements references

1. Abarbanel, B.; Gainsbury, S.M.; King, D.; Hing, N.; Delfabbro, P.H. Gambling games on social platforms: How do advertisements for social casino games target young adults? *Policy Internet* **2017**, *9*, 184–209.
2. Baloglu, S.; Zhong, Y.Y.; Tanford, S. Casino loyalty: The influence of loyalty program, switching costs, and trust. *J. Hosp. Tour Res.* **2017**, *41*, 846–868.
3. Gainsbury, S.M.; King, D.L.; Hing, N.; Delfabbro, P. Social media marketing and gambling: An interview study of gambling operators in Australia, *Int. Gambl. Stud.* **2015**, *15*, 377–393.
4. Gainsbury, S.M.; Delfabbro, P.; King, D.L.; Hing, N. An exploratory study of gambling operators' use of social media and the latent messages conveyed. *J. Gambl. Stud.* **2016**, *32*, 125–141.
5. Gainsbury, S.M.; King, D.L.; Russell, A.M.T.; Delfabbro, P.; Derevensky, J.; Hing, N. Exposure to and engagement with gambling marketing in social media: Reported impacts on moderate-risk and problem gamblers. *Psychol Addict. Behav* **2016**, *30*, 270–276.
6. Hing, N.; Sproston, K.; Brook, K.; Brading, R. The structural features of sports and race betting inducements: Issues for harm minimisation and consumer protection. *J. Gambl. Stud.* **2017**, *33*, 685–704.
7. Hing, N.; Russell, A.M.T.; Li, E.; Vitartas, P. Does the uptake of wagering inducements predict impulse betting on sport? *J. Behav. Addict.* **2018**, *7*, 146–157.
8. O'Loughlin I, Blaszczynski A. Comparative effects of differing media presented advertisements on male youth gambling attitudes and intentions. *Int J. Mental Health Ad* **2018**, *16*, 313–327.
9. Prentice, C.; Wong, I.A. Casino marketing, problem gamblers or loyal customers? *J. Bus. Res.* **2015**, *68*, 2084–2092.
10. Quigno, J.; Zhang, L. Casino customers' intention to join a loyalty rewards program: The effect of number of tiers and gender. *Cornell Hosp. Quart* **2016**, *57*, 226–230.
11. Yoo, M.; Singh, A. Comparing loyalty program tiering strategies: An investigation from the gaming industry. *UNLV Gaming Res. Rev. J.* **2016**, *20*, 19–40.
